# Emergence of Progressive Mutations in SARS-CoV-2 From a Hematologic Patient With Prolonged Viral Replication

**DOI:** 10.3389/fmicb.2022.826883

**Published:** 2022-03-03

**Authors:** Carolina Garcia-Vidal, María Iglesias-Caballero, Pedro Puerta-Alcalde, Vicente Mas, Genoveva Cuesta-Chasco, Nicole Garcia-Pouton, Sarai Varona, Francisco Pozo, Sonia Vázquez-Morón, Maria Angeles Marcos, Alex Soriano, Inmaculada Casas, HEMATOCOVID19-Researchers Group

**Affiliations:** ^1^Department of Infectious Diseases, Hospital Clinic of Barcelona-IDIBAPS, University of Barcelona, Barcelona, Spain; ^2^Respiratory Virus and Influenza Unit, National Center of Microbiology, National Influenza Center, Instituto de Salud Carlos III, Majadahonda, Madrid, Spain; ^3^Department of Microbiology, ISGlobal, Hospital Clinic-Universitat de Barcelona, Barcelona, Spain; ^4^Bioinformatics Unit, Instituto de Salud Carlos III, Majadahonda, Madrid, Spain; ^5^Centro de Investigación Biomédica en Red de Epidemiología y Salud Pública (CIBERESP), Madrid, Spain

**Keywords:** COVID-19, antivirals, persistence, mutations, remdesivir, immunosuppression, hematology

## Abstract

We documented a hematologic patient with prolonged SARS-CoV-2 viral replication in whom emergence of viral mutations was documented after the consecutive use of antivirals and convalescent plasma. The virus detected in the last of 12 clinical samples (day 237) had accumulated 22 changes in amino acids and 29 in nucleotides. Some of these changes, such as the E484Q, were mutations of concern as defined by WHO. This finding represents an enormous epidemiological threat and poses a major clinical challenge. Combined antiviral strategies, as well as specific strategies related to the diagnostic approach of prolonged infections for this specific population, may be needed.

## Introduction

Severe acute respiratory syndrome coronavirus-2 (SARS-CoV-2) is a novel coronavirus first identified in Wuhan, China during December 2019 (Zhu et al., 2020). Responsible for coronavirus disease COVID-19, SARS-CoV-2 triggered a pandemic that would cause more than 4 million deaths and pose a threat to global health [WHO Coronavirus (COVID-19) Dashboard|WHO Coronavirus (COVID-19), 2021 Dashboard With Vaccination Data].

Indeed, a major issue that has arisen in this health crisis has concerned the emergence of breakthrough viral variants following virus mutations. Such mutations in the spike protein may confer a higher affinity for the cell receptor leading to a higher viral load in respiratory secretions and modifying the critical epitopes used to develop vaccines (Miller et al., 2021). The consequences are the rapid selection of these variants with a significant impact on transmissibility, severity, and/or immunity and, more challenging, the reduction of the efficacy of current vaccines (Hodcroft et al., 2021).

Mutation rate in the *Coronaviridae* family is not high, with a replication error rate more than 10-fold lower than that of other RNA viruses (Rausch et al., 2020). However, two circumstances may favor the emergence of new variants. First, an uncontrolled pandemia with millions of people infected, and second, when the immune system of the infected host is not able to control the virus. The second scenario is particularly concerning because potential resistant virus could be selected due to the pressure exerted by antivirals, convalescent plasma, or monoclonal antibodies especially when administered sequentially and because there is a risk to disseminate this new virus to the community (Avanzato et al., 2020; Choi et al., 2020).

We aimed to describe the clinical characteristics of an immunocompromised patient with persistent shedding of SARS-CoV-2 during the follow-up period, demonstrated by genomic and sub-genomic RNA characterization and positive viral culture. We describe the genetic sequence of detected viruses showing that all of them corresponded to the same virus, the acquisition of mutations, and the potential influence of the different treatments that the patient received.

## Materials and Methods

The patient-in-question received care at Hospital Clinic in Barcelona (Spain). All positive samples were tested for the presence of sub-genomic RNA (sgRNA). Genomic studies of SARS-CoV-2 were done at the Respiratory Virus and Influenza Unit, National Center of Microbiology (ISCIII). A thorough description of microbiological studies, sequencing and bioinformatic methods are provided in the Supplementary Material.

## Results

### Description of Patient

**Figure 1:**
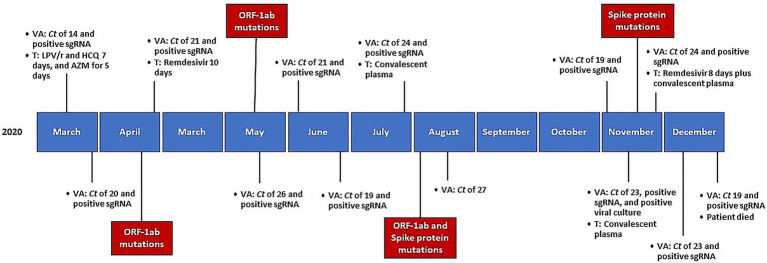
Timeline of microbiological testing, received treatments, and mutations emergence, since initial diagnosis. VA, viral assessment; Ct, cycle threshold; sgRNA, sub-genomic RNA; T, treatment; LPV/r, lopinavir/ritonavir; HCQ, hydroxychloroquine; AZM, azithromycin.

Female patient with diffuse large B-cell lymphoma (DLBCL) stage IV-B that received treatment with six cycles of R-CHOP. The most recent cycle of R-CHOP was 6 months before the first COVID-19 hospital admission. In February 2020, the patient relapsed and began palliative care with cyclophosphamide and prednisone (PDN) cycles. On March 2020, she was admitted to the hospital for bilateral pneumonia with a total lymphocyte count of 200 cells/μL. SARS-CoV-2 RT-PCR was performed on a nasopharyngeal swab (NPS), testing positive with a Ct value of 14. The patient received lopinavir/ritonavir (LPV/r) and hydroxychloroquine (HCQ) for 7 days and azithromycin (AZM) for 5 days, since it was the standard therapy at that time at our center, although is no longer recommended. On day +20, she was discharged without control RT-PCR testing. However, she presented a persistent, low-grade fever attributed to tumor progression and underwent treatment with PDN. On day +52, she was re-admitted to our hospital due to a persistent cough and worsening of fever. A chest X-ray showed persistence of bilateral pneumonia. RT-PCR of NPS tested positive for SARS-CoV-2, with a Ct value of 21 and positive sgRNA. SARS-CoV-2 serology was negative. An extensive work-up ruled out other possible causes of fever, and the patient received remdesivir for 10 days. The patient’s health improved progressively, and she was discharged. Despite this treatment, RT-PCR of NPS remained positive and there were no significant changes in Ct values and sgRNA was positive. Receiving care on an outpatient basis, the patient presented a persistent dry cough and persistently positive RT-PCR of NPS. On day +139, she was re-admitted to our hospital for further study. A computed tomography (CT) scan showed persistence of pulmonary infiltrates. Serology remained negative. A new RT-PCR of NPS tested positive, with a Ct value of 24 and positive sgRNA once again. She received convalescent plasma and was then discharged. The patient presented mild symptoms, but RT-PCR of NPS remained positive while she received care on an outpatient basis. Eight months after the initial hospitalization, the patient was re-admitted again for COVID-19 evaluation. A chest CT showed mild ground-glass opacities (without significant changes). The serological analysis showed negative results, and again RT-PCR of NPS tested positive with a Ct value of 23 and positive sgRNA. At this point, isolation of SARS-CoV-2 was made from a NPS of in cell culture at +146 days. The patient then received a new dose of convalescent plasma. A month later, a febrile neutropenic episode elicited re-admission of the patient. RT-PCR of NPS tested positive again, with a Ct value of 24 and positive sgRNA. After these findings, the patient underwent a second course of remdesivir for 8 days concomitantly with a new dose of convalescent plasma. At the end of treatment, RT-PCR of NPS tested positive (Ct value of 19), as did sgRNA and the viral culture. The patient died in December due to lymphoma progression. Figure 1 details the timeline of microbiological testing and treatments of this patient.

### Genetic Study of SARS-CoV-2 From Clinical Samples

The patient presented an extended period of sampling (237 days), in which 12 samples were available for genetic analysis. Before the analysis of mutations over time, to discard the possibility of SARS-CoV-2 re-infection was a need. The assignation of B.1 lineage using Pango lineage assignment tool in every sample from the patient, was the first clue to exclude a re-infection. The patient presented 6 nucleotide mutations and 2 amino acid changes from the reference sequence Wuhan-1 (GenBank accession number: NC_045512): C96T, C241T, G12769T, C14408T, A20268G, A23403G, being present in the 12 consensus sequences analyzed from the patient. According to the A20268G mutation, the nucleotide change produces a stop codon lost for a tryptophan amino acid. An analysis of all B.1 sequences published in Spain from March to December 2020 revealed the presence of this change only in the 27% of the samples (Supplementary Figure S1). Supplementary Figure S2 shows the phylogenetic tree based on the analysis of B.1 complete sequences published in GISAID from Spain from March to December 2020. GISAID accession numbers for the sequences included in the tree are listed in the Supplementary Table S1. Supplementary Table S2 summarizes the most important aspects of the genetic study. After the entire study period, in the last NPS sample the virus accumulated 29 mutations in nucleotides and 22 amino acid changes.

### Genomic Analysis Over Time

#### Changes in ORF1ab Polyprotein

Analysis of the ORF1ab polyprotein revealed an accumulation of mutations over time. Focusing on the minor variant analysis, the presence of viral subpopulations in each ORF1ab protein was detected from July onward, especially in four domains of the non-structural protein 3 (nsp3): Mac1, Mac 2, NAB, and UBl2-Pl2^pro^. Since the initial samples, 6 different amino acids appeared: A1105V, T540I, K977Q, S370L, E746A, and T820I. Supplementary Figure S3 detailed mutations over time.

#### Changes in the Spike

**Figure 2:**
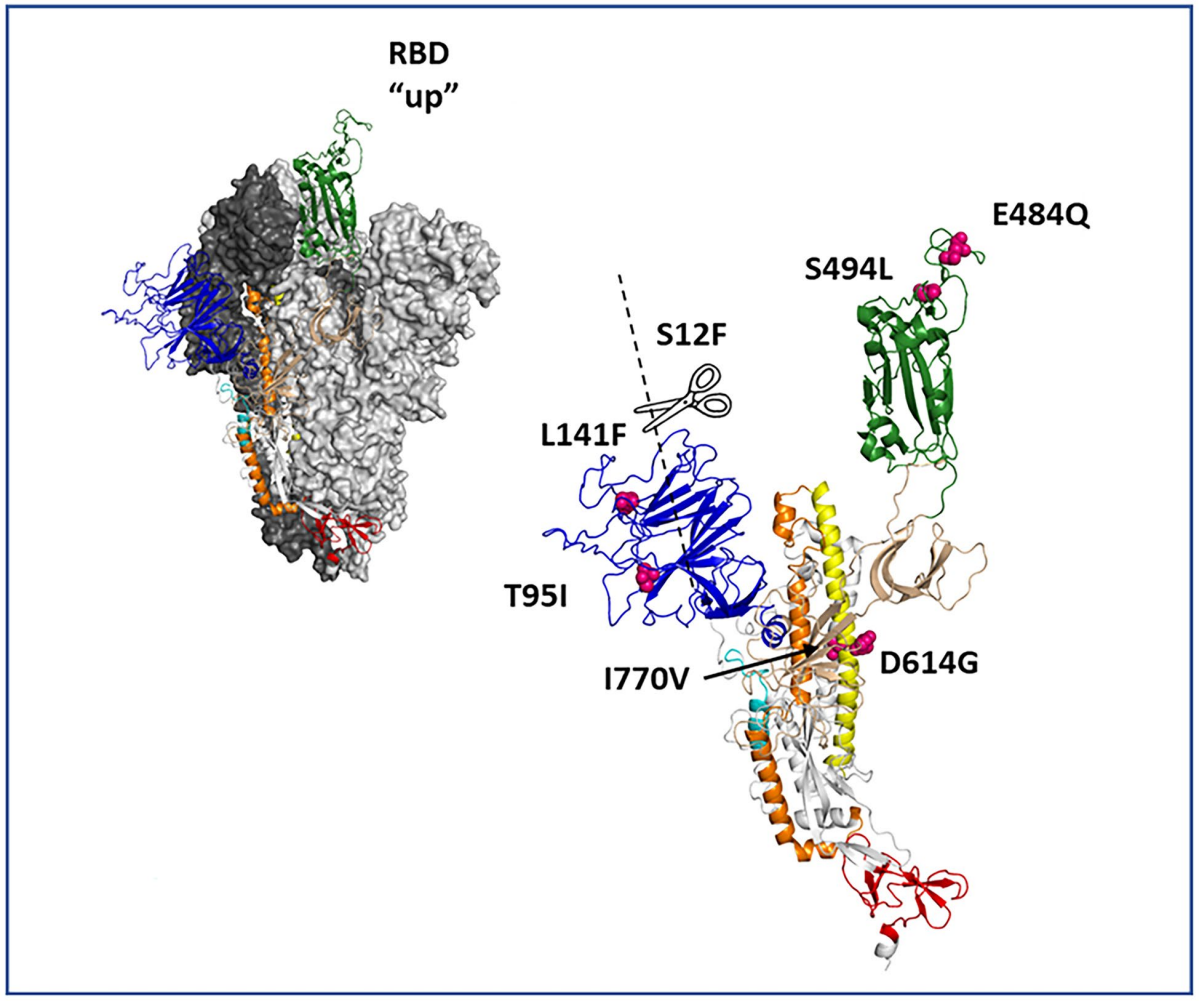
Protein depiction of the spike homotrimer in open conformation. The residues involved in amino acid substitutions are pointed in the structure depiction.

Mutation D614G was found in the first virus analyzed in accordance with the lineage B.1. Cumulative changes were documented over time. Patterns of variant frequency suggested competition between viral populations carrying different mutations. Specifically, two events related to the emergence and re-emergence of three changes (T95I, S494L, and Δ143/144 or Δ143/145) were identified in virus from samples from days 128 and 237. These changes were subsequently reversed in the following up virus. Remarkable, mutation E484Q was documented from day 132 onward. Supplementary Figure S4 detailed the most important changes in consensus sequences of the Spike over time. Supplementary Figure S5 showed the 7 amino acid changes in the Spike of virus detected in the last sample: S12F, T95I, L141F, E484Q, S494L, D614G, and I770V. The Spike structure showed a signal peptide mutation, S12F, and two changes in the N-terminal domain (NTD), T95I and L141F. Also, two changes were identified in the receptor-binding domain (RBD): E484Q and S494. Figure 2 showed the location of the Spike changes.

Analysis of minority variants population revealed the presence of mutations T95I and S494L as viral subpopulations with a frequency <25%. Mutation I770V reached almost 75% of the viral population in the sample taken from day 128 and 100% in the virus from sample collected on day 237.

## Discussion

This study documented the evolution of SARS-CoV-2 by progressive acquisition of mutations during the course of a persistent infection in an immunocompromised patient with a lymphoproliferative disease and rituximab treatment who received different antiviral strategies. The detected mutations were located in different sites, including the viral Spike, with troubling repercussions for the patient, given their ability to evade immunity.

Prolonged viral replication has been documented in immunocompromised hosts (Avanzato et al., 2020; Wölfel et al., 2020; Young et al., 2020), with little information focusing on genetic variations. Avanzato et al. reported a genetic analysis of four samples from an asymptomatic patient (Avanzato et al., 2020), in which the authors documented variations in the viral genome over time with no impact on replication kinetics. Their findings suggested the co-existence of minor variants within the same host. In our work, we confirmed the evolution of virus detecting minor variants and detailed cumulative mutations after antiviral treatment strategies.

Our patient initially received LPV/r and later remdesivir and convalescent plasma consecutively. The anti-HIV protease LPV/r may inhibit the action of the SARS-CoV-2 3CLpro proteinase (Uzunova et al., 2020), and its use may have played a role in the nsp3 mutations.

Remdesivir inhibits SARS-CoV-2 RNA replication by two mechanisms that target ORF-1ab, more specifically, nsp12 (Gordon et al., 2020; Tchesnokov et al., 2020). The virus developed different ORF-1ab mutations, albeit all were located in the nsp3 gene. This protein has a major role in viral replication although it was not the target of any of the treatments administered to the patient. Of note, nsp3 includes the essential domain of SARS-CoV-2 protein PL2pro that plays a significant role in how the virus interacts with the innate immune system (Angeletti et al., 2020). We also identified the T820I and K977Q mutations representing a change in the enzyme substrate preference diminishing the host’s ability to recognize the virus. Both mutations were related with ubiquitin-like ISG15 host protein interaction. A glutamine in the 977 position was observed in the 2003 SARS-CoV Urbani virus strain (GenBank accession number: AY278741.1) suggesting that this change might be represented in commonly circulating *Sarbecovirus* with particularly affinity to evade immune system.

Treatment with convalescent plasma was administered in July and November 2020. After that, progressive amino acid mutations in the spike protein were documented. Those cumulative mutations could affect the effectiveness of neutralizing antibodies. The spike structure showed different changes: (i) a signal peptide mutation, S12F; (ii) two changes in the N-terminal domain, T95I and L141F; and (iii) two changes in the receptor-binding domain, E484Q and S494. Due to the location of these changes, viral affinity for ACE-2 receptor and interaction with neutralizing antibodies could be compromised. Mutation T95I is also present in many Delta variants of concern (VOC) sub-lineages and in the Omicron VOC (European Centre for Disease Prevention and Control, 2021). Mutation E484Q was first identified in variant Kappa (B.1.617.1) and in B.1.617.3 lineage (European Centre for Disease Prevention and Control, 2021). This mutation was defined as a mutation of concern by the WHO based on changes in the antibodies binding site Ib in the receptor-binding domain. Also, the mutation E484Q present in our patient is very similar to the 484A present in the Omicron VOC (European Centre for Disease Prevention and Control, 2021). Deletions detected, Δ143/145 and Δ143/144, located in the N-terminal domain, are considered recurrent deletions that present a disruption to antibody binding (McCarthy et al., 2021), being the Δ143/145 a deletion of interest of the Alpha VOC (European Centre for Disease Prevention and Control, 2021). Since the patient did not present seroconversion, convalescent plasma could have played a role in the emergence of such mutations in the spike protein.

The results detailed in our study are highly significant in clinical practice. Physicians treating severely immunocompromised patients should be aware of this potentially prolonged viral replication. Both the patient and overall public health face the risk of serious consequences. For instance, the patient may be ill for an extended time, thereby causing delays in medical care needed for baseline diseases. Additionally, the selection of virus able to escape from the immune system and neutralizing activity of antibodies generated by vaccines, or those with specific mutations that could confer resistance to antivirals may limit the capacity to control the pandemic. Implementing an aggressive approach that aims to diminish viral load at disease onset and employing appropriate public health actions appears to be of the utmost importance. Guidelines on SARS-CoV-2 treatment and transmission should outline specific recommendations for immunocompromised patients. Since RT-PCR targeting SARS-CoV-2 genes are not well related to virus viability (Zou et al., 2020), it may be recommendable to consider the use of RT-PCR in identifying viral sub-genomic mRNAs, which is better correlated with active viral replication (Santos Bravo et al., 2021), or viral culture as a follow-up criterion.

The strengths of this study include complete clinical information and viral genetic details of a high number of consecutive samples taken from the same patient for a long period of time. However, there are some limitations concerning the clarification of viral persistence mechanisms. Neutralization studies *in vitro* were not performed. Moreover, culture was not performed in all samples and the clinical implications of each mutation deserve further studies.

In summary, sustained SARS-CoV-2 replication in immunosuppressed patients occurs. We documented potential resistant virus selected due to the pressure exerted by progressive use of convalescent plasma and antivirals. This finding represents an enormous epidemiological threat and poses a major impact and a clinical challenge. Extended and combined antiviral strategies, as well as specific strategies related to decisions regarding isolation measures for this specific population may be needed.

## Data Availability Statement

The original contributions presented in the study are included in the article/Supplementary Material, further inquiries can be directed to the corresponding author.

## Ethics Statement

The Institutional Ethics Committee of Hospital Clinic of Barcelona approved the study and waived the need for informed consent from individual patients due to the nature of the retrospective data review (HCB/2020/0273).

## Author Contributions

CG-V, PP-A, MM, and AS: conceptualization. CG-V, PP-A, AS, and IC: methodology. MI-C, GC-C, FP, SV, SV-M, and IC: software. CG-V, MI-C, AS, and IC: formal analysis. PP-A, MI-C, CG-V, and IC: investigation. CG-V, MI-C, PP-A, VM, GC-C, NG-P, SV, FP, SV-M, MM, AS, and IC: resources, data curation, writing—review and editing, and visualization. CG-V, MI-C, PP-A, AS, and IC: writing—original draft. CG-V, IC, and AS: supervision. PP-A and CG-V: project administration. All authors contributed to the article and approved the submitted version.

## Funding

This work has been financed by funds for research *ad hoc* COVID-19 from patronage provided by citizens and organizations to Hospital Clínic de Barcelona-Fundació Clínic per a la Recerca Biomèdica. This work received support from FONDO-COVID19 (ISCIII Grant number: COV20-00679), Instituto de Salud Carlos III (PI21/01640) and by European Region (ERDF, “A way to make Europe”). PP-A [JR20/00012 and PI21/00498], NG-P [FI19/00133], have also received research grants from the Ministerio de Sanidad y Consumo, Instituto de Salud Carlos III. The funders had neither a specific role in study design or collection of data, nor in writing of the paper or decision to submit.

## Conflict of Interest

CG-V has received honoraria for talks on behalf of Gilead Science, MSD, Novartis, Pfizer, Janssen, Lilly as well as a grant from Gilead Science and MSD. AS has received honoraria for talks on behalf of Merck Sharp and Dohme, Pfizer, Novartis, Angelini, as well as grant support from Pfizer. PP-A has received honoraria for talks on behalf of Merck Sharp and Dohme, Gilead, Lilly, ViiV Healthcare, and Gilead Science.

The remaining authors declare that the research was conducted in the absence of any commercial or financial relationships that could be construed as a potential conflict of interest.

## Publisher’s Note

All claims expressed in this article are solely those of the authors and do not necessarily represent those of their affiliated organizations, or those of the publisher, the editors and the reviewers. Any product that may be evaluated in this article, or claim that may be made by its manufacturer, is not guaranteed or endorsed by the publisher.
