# Supplementary material for: Emergence of Progressive Mutations in SARS-CoV-2 From a Hematologic Patient With Prolonged Viral Replication
Source: Front Microbiol. 2022 Mar 3;13:826883. doi: 10.3389/fmicb.2022.826883 (PMC8927661; doi:10.3389/fmicb.2022.826883)
Supplement: Supplementary file 1 [file Data_Sheet_1.pdf]

Supplementary material. Emergence of progressive mutations in a hematologic patient with SARS-CoV-2 and prolonged viral replication.

Supplementary methods.

Microbiological studies

Reverse transcription polymerase chain reaction (RT-PCR) was performed using Cobas®6800 (Roche Diagnostic, Germany), which detects E and ORF1b genes. A positive result was defined when the cycle threshold (Ct) value of the E gene was ≤ 38. All positive samples were tested for the presence of sub-genomic RNA (sgRNA) with primers and probes targeting sequence downstream of the start codons of the E gene, as described with a specific forward leader primer (Wölfel R. et al. *Nature* 2020. doi:10.1038/s41586-020-2196-x). sgRNA was performed using the SuperScript™ III Platinum™ One-Step qRT-PCR Kit (Invitrogen) in the thermocycler StepOne (Applied Biosystems). A positive result was defined when the Ct value of the E gene was ≤ 39.

For viral culture, each sample was treated with a mixture of antibiotics (vancomycin and streptomycin) and antifungal (amphotericin B) for 30 minutes. After sample treatment, 300 µl were inoculated into the VERO-E6 cell line and incubated at 37°C for a maximum of 10 days. A culture was considered positive when a characteristic CPE was observed. The CPE was confirmed to be caused by SARS-CoV-2 by indirect immunofluorescence using a specific monoclonal antibody AntiSARS-CoV-2 (CERTEST, Spain). The viral culture was considered negative in the absence of CPE 10 days after inoculation.

Sequencing methods

Whole genome amplification for NGS sequencing was performed at the National Center of Microbiology (ISCIII). Sample cDNAs were obtained by means of a Sequence-Independent, Single-Primer-Amplification (SISPA) using the random primer FR26RV-N (5'-GCCGGAGCTCTGCAGATATCNNNNNN-3') (Pérez-Sautu u. et al. *Emerg Microbes Infect* 2019. doi:10.1080/22221751.2019.1640587). Subsequently, cDNA products were amplified following ARTIC network's PCR protocol, with the primer pool version "ARTIC n-CoV-2019 v3" (Ewels P. et al. *Bioinformatics* 2016. doi:10.1093/bioinformatics/btw354). Libraries were performed following Nextera DNA library preparation kit instructions (Illumina, California, USA). These libraries were barcoded with unique dual indexes and then pooled and sequenced using a NextSeq Reagent kit 300 cycles (Illumina, California, USA) in a NextSeq sequencer (Illumina, California, USA). The sequences are available in the GISAID database (Shu Y. et al. *Eurosurveillance* 2017. doi:10.2807/1560-7917.ES.2017.22.13.30494).

To confirm Spike sequences, two PCRs targeting the S1 and S2 regions of the Spike protein were performed. The primers used in amplifying these regions included ARTIC pool primers 71L and 78R for S1 region and 77L and 84R primers for S2 region. Amplification conditions were: RT at 48°C - 20 min, 95°C - 5 min, followed by 45 cycles, 95°C - 15 s, 64°C - 2 min, 68°C - 30 s. Amplified products (~2490 bp S1 and ~2551 bp S2) were visualized performing an electrophoresis on a 1% agarose gel. Purified, amplified products of the expected size were double-strand sequenced by Sanger chain-termination method, using the BigDye Terminator v3.1 Cycle Sequencing Kit in an ABI PRISM 3700 DNA Analyzer (Applied Biosystems).

Quality control of the consensus sequences was based on the evaluation of the number of Ns in the consensus genome; number of ambiguous bases (Non A, T, C, or G): presence of mutations that only occur in this particular consensus genome; and description of frameshifts and premature stop codons. According to this quality control, the status was good in 10 of the 12 sequences analyzed, with a mean depth of coverage obtained between 4000-6000x.

With respect to the good-quality consensus sequences, 9 of these 12 samples had genome coverage superior to 90% after whole genome sequencing (WGS). In samples in which genome coverage was between 70-90%, genomic fragments negative by NGS methods were re-amplified and sequenced by Sanger. Spike genes in all samples were also sequenced by Sanger to confirm specific mutations.

Bioinformatic methods

Sequencing samples were analyzed for viral consensus genome reconstruction using the viralrecon pipeline (https://github.com/nf-core/viralrecon) written in Nextflow (https://www.nextflow.io/) in collaboration with the nf-core (https://nf-co.re/) community and the Bioinformatics Unit of the Institute of Health Carlos III (BU-ISCIII) (https://github.com/BU-ISCIII). In this pipeline, fastq files containing raw reads were first analyzed for quality using FastQC v0.11.9 (http://www.bioinformatics.babraham.ac.uk/projects/fastqc/). Raw reads were trimmed using fastp v.0.20.1 (Chen S. et al. *Bioinformatics* 2018. doi:10.1093/bioinformatics/bty560). The sliding window quality filtering approach was performed, scanning the read with a 4-base-wide sliding window and cutting 3’ and 5’ ends of the base when average quality per-base dropped below a Qphred33 of 30.

Reads shorter than 50 nucleotides and reads with more than 10% of read quality under Qphred 30 were removed. Additionally, poly-X sequenced were removed from read ends. Trimmed reads were mapped against the reference SARS-Cov2 genome (NC_045512.2) with bowtie2 v.2.3.5.1 (Langmead B. et al. *Nat Methods* 2012. doi:10.1038/nmeth.1923). Amplicon primers were then soft-clipped from mapping files using iVar v.1.2.2 (Grubaugh ND. et al. *Genome Biol* 2019. doi:10.1186/s13059-018-1618-7). Picard v.2.22.8 (https://github.com/broadinstitute/picard) and SAMtools v.1.9 (Li H. et al. *Bioinformatics* 2019. doi:10.1093/bioinformatics/btp352), were used to generate viral genome mapping stats. To obtain statistics about host genome content, kmer-based mapping of the trimmed reads against the GRCh38 NCBI human genome reference was performed with Kraken2 v.2.0.9beta (Wood DE. et al. *Genome Biol* 2019. doi:10.1186/s13059-019-1891-0).

Variant calling was done using VarScan2 v.2.4.4 (Koboldt DC. et al. *Bioinformatics* 2009. doi:10.1093/bioinformatics/btp373), which calls for low and high frequency variants from which variants with an allele frequency higher than 80 were kept to be included in consensus genome sequence. Both, variants included and not included in the consensus genome sequence were annotated using SnpEff v.4.5covid19 (Cingolani P. et al. *Fly (Austin)* 2012. doi:10.4161/fly.19695), and SnpSift v.4.3.1t (Cingolani P. et al. *Front Genet* 2012. doi:10.3389/fgene.2012.00035). Finally, bedtools v2.29.2 (Quinlan AR. et al. *Bioinformatics* 2010. doi:10.1093/bioinformatics/btq033) was used to obtain the viral genome consensus with the filtered variants and mask genomic regions with coverage values lower than 10X. Final summary reports were created using MultiQC v.1.9 (Ewels P. et al. Bioinformatics 2016. doi:10.1093/bioinformatics/btw354).

The variant calling files (vcf) obtained with VarScan2 containing all of the variants (low and high frequency) were analyzed with Bcftools v.1.10.2 to merge all of the samples and select the allele frequency field (Danecek P. et al. *Gigascience* 2021. doi:10.1093/gigascience/giab008). Annotation information from SnpEff was then added to the variants. In order to create ad-hoc tables and allele frequency evolution plots, the resulting table underwent further processing with R v.3.6.1.

Supplementary results.

Supplementary Figure S1. Aminoacidic alignment chart of the patient's sequences with all the B.1 complete sequences published in Spain from March to December 2020 (n= 6761 sequences).

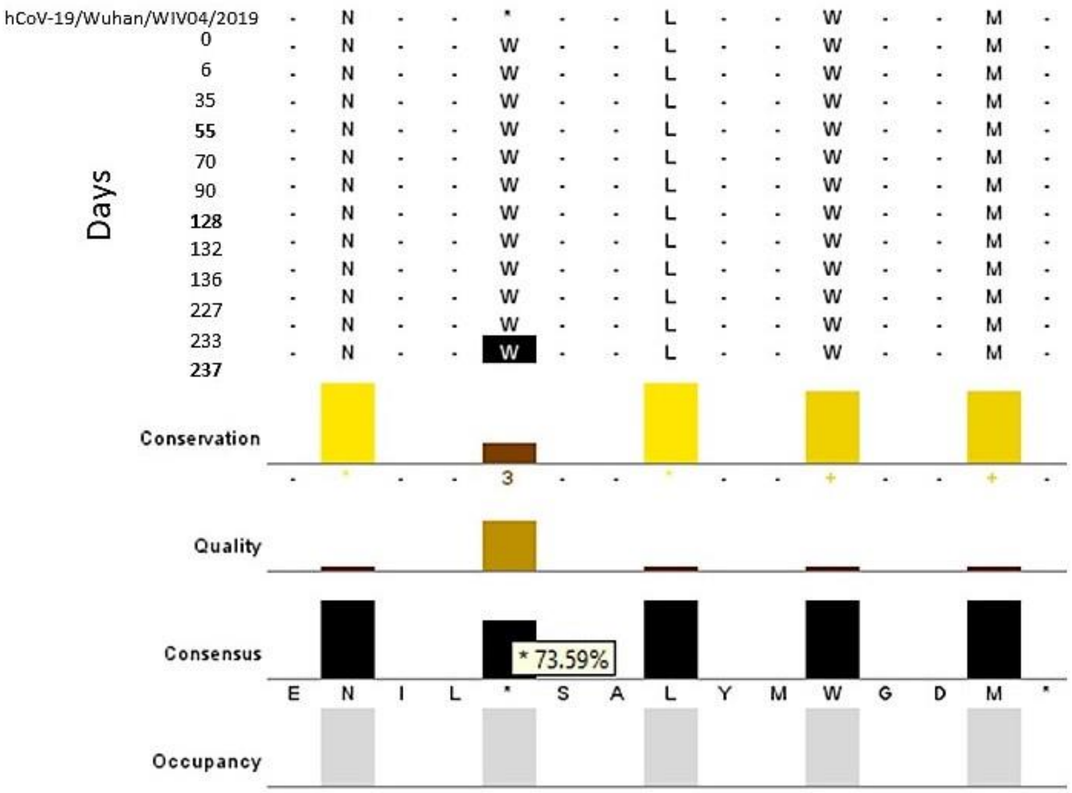

Legend. The presence of a Tryptophan in the position 6668 occurred 27% of the times.

Supplementary Figure S2. Phylogenetic tree based on the analysis of B.1 complete sequences published in GISAID from Spain from March to December 2020 (n= 741 sequences).

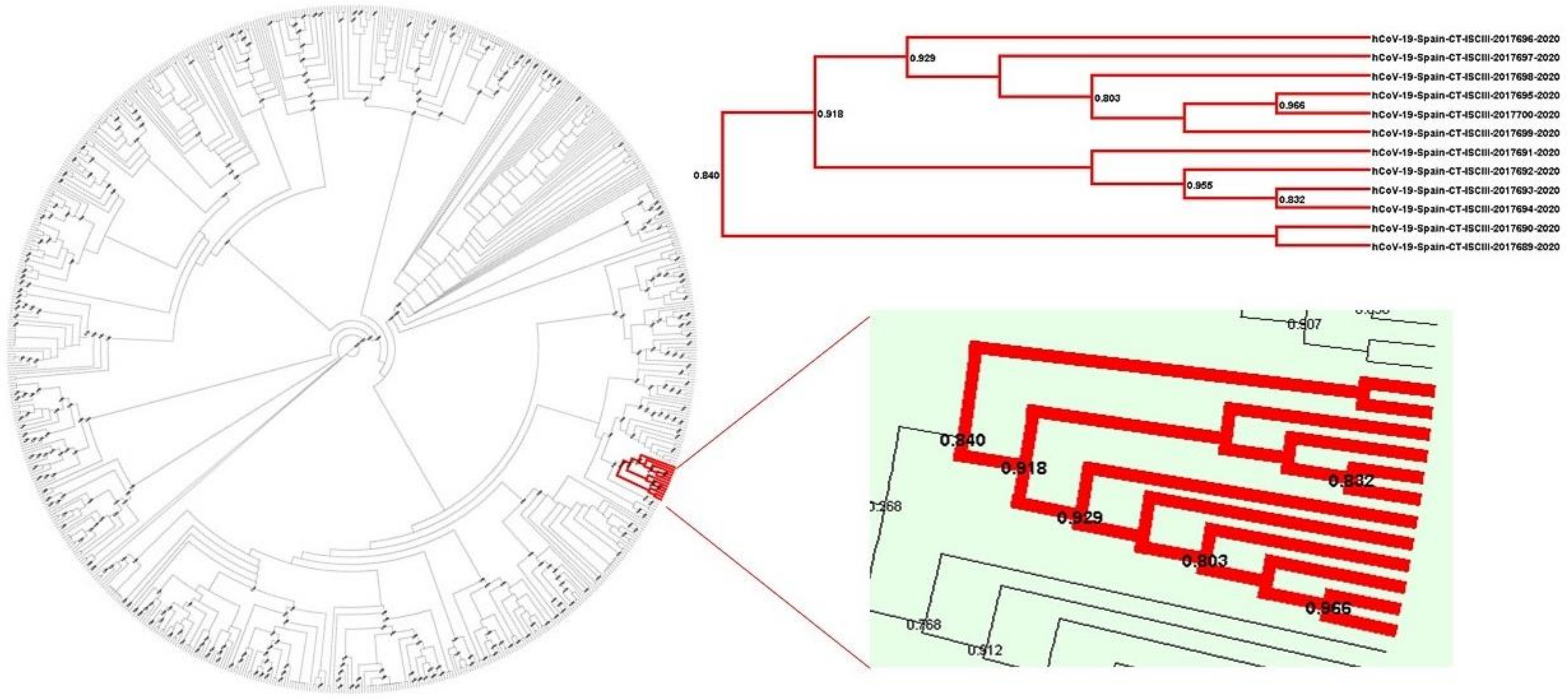

Legend. Trees were estimated with FastTreeMP software by using the Maximum Likelihood method on Generalized Time-Reversable model. A bootstrap test was replicated for 1000 times. Numbers represent percentage bootstrap support. GISAID accession numbers for the sequences included in the tree are listed in Supplementary Table S1.

Supplementary Table S1. GISAID accession numbers from the sequences used in the phylogenetic analysis.

Sequence ID	GISAID accession number
hCoV-19-Spain-VC-FISABIO-187-2020	EPI-ISL-436282
hCoV-19-Spain-VC-FISABIO-192-2020	EPI-ISL-436287
hCoV-19-Spain-VC-FISABIO-202-2020	EPI-ISL-436297
hCoV-19-Spain-AR-IBV-2451-2020	EPI-ISL-468770
hCoV-19-Spain-AR-IBV-2487-2020	EPI-ISL-468771
hCoV-19-Spain-AR-IBV-2414-2020	EPI-ISL-468772
hCoV-19-Spain-AR-IBV-2475-2020	EPI-ISL-468775
hCoV-19-Spain-AS-IBV-98006633-2020	EPI-ISL-654625
hCoV-19-Spain-AS-IBV-98006624-2020	EPI-ISL-654627
hCoV-19-Spain-AS-IBV-98006651-2020	EPI-ISL-654629
hCoV-19-Spain-AS-IBV-98006628-2020	EPI-ISL-654631
hCoV-19-Spain-AS-IBV-98006646-2020	EPI-ISL-654633
hCoV-19-Spain-VC-FISABIO-106-2020	EPI-ISL-436201
hCoV-19-Spain-VC-FISABIO-108-2020	EPI-ISL-436203
hCoV-19-Spain-VC-FISABIO-110-2020	EPI-ISL-436205
hCoV-19-Spain-VC-FISABIO-112-2020	EPI-ISL-436207
hCoV-19-Spain-VC-FISABIO-114-2020	EPI-ISL-436209
hCoV-19-Spain-VC-FISABIO-129-2020	EPI-ISL-436224
hCoV-19-Spain-VC-FISABIO-134-2020	EPI-ISL-436229
hCoV-19-Spain-VC-FISABIO-135-2020	EPI-ISL-436230
hCoV-19-Spain-VC-FISABIO-142-2020	EPI-ISL-436237
hCoV-19-Spain-VC-FISABIO-145-2020	EPI-ISL-436240
hCoV-19-Spain-VC-FISABIO-147-2020	EPI-ISL-436242
hCoV-19-Spain-VC-FISABIO-149-2020	EPI-ISL-436244
hCoV-19-Spain-VC-FISABIO-150-2020	EPI-ISL-436245
hCoV-19-Spain-VC-FISABIO-152-2020	EPI-ISL-436247
hCoV-19-Spain-VC-FISABIO-155-2020	EPI-ISL-436250
hCoV-19-Spain-VC-FISABIO-157-2020	EPI-ISL-436252
hCoV-19-Spain-VC-FISABIO-163-2020	EPI-ISL-436258
hCoV-19-Spain-VC-FISABIO-166-2020	EPI-ISL-436261
hCoV-19-Spain-VC-FISABIO-167-2020	EPI-ISL-436262
hCoV-19-Spain-VC-FISABIO-172-2020	EPI-ISL-436267
hCoV-19-Spain-VC-FISABIO-173-2020	EPI-ISL-436268
hCoV-19-Spain-VC-FISABIO-176-2020	EPI-ISL-436271
hCoV-19-Spain-VC-FISABIO-177-2020	EPI-ISL-436272
hCoV-19-Spain-VC-FISABIO-156-2020	EPI-ISL-436251
hCoV-19-Spain-AR-IBV-2387-2020	EPI-ISL-468840
hCoV-19-Spain-AR-IBV-2419-2020	EPI-ISL-468842
hCoV-19-Spain-AR-IBV-2430-2020	EPI-ISL-468843
hCoV-19-Spain-AR-IBV-2396-2020	EPI-ISL-468851
hCoV-19-Spain-AR-IBV-2462-2020	EPI-ISL-468854
hCoV-19-Spain-NC-CHN-01000278-2020	EPI-ISL-1382967
hCoV-19-Spain-NC-CHN-01000280-2020	EPI-ISL-1382968
hCoV-19-Spain-NC-CHN-01000281-2020	EPI-ISL-1382969
hCoV-19-Spain-NC-CHN-01000282-2020	EPI-ISL-1382970
hCoV-19-Spain-NC-CHN-01000285-2020	EPI-ISL-1382972
hCoV-19-Spain-NC-CHN-01000286-2020	EPI-ISL-1382973
hCoV-19-Spain-NC-CHN-01000288-2020	EPI-ISL-1382975
hCoV-19-Spain-NC-CHN-01000289-2020	EPI-ISL-1382976
hCoV-19-Spain-NC-CHN-01000291-2020	EPI-ISL-1382978
hCoV-19-Spain-NC-CHN-01000293-2020	EPI-ISL-1382980
hCoV-19-Spain-NC-CHN-01000297-2020	EPI-ISL-1382984
hCoV-19-Spain-NC-CHN-01000300-2020	EPI-ISL-1382986
hCoV-19-Spain-NC-CHN-01000301-2020	EPI-ISL-1382987
hCoV-19-Spain-NC-CHN-01000302-2020	EPI-ISL-1382988
hCoV-19-Spain-NC-CHN-01000303-2020	EPI-ISL-1382989
hCoV-19-Spain-NC-CHN-01000305-2020	EPI-ISL-1382991
hCoV-19-Spain-NC-CHN-01000306-2020	EPI-ISL-1382992
hCoV-19-Spain-NC-CHN-01000308-2020	EPI-ISL-1382993
hCoV-19-Spain-NC-CHN-01000311-2020	EPI-ISL-1382996
hCoV-19-Spain-NC-CHN-01000312-2020	EPI-ISL-1382997
hCoV-19-Spain-NC-CHN-01000314-2020	EPI-ISL-1382999
hCoV-19-Spain-AR-IBV-2428-2020	EPI-ISL-468805
hCoV-19-Spain-AR-IBV-2479-2020	EPI-ISL-468813
hCoV-19-Spain-AR-IBV-2422-2020	EPI-ISL-468815
hCoV-19-Spain-AR-IBV-2412-2020	EPI-ISL-468858
hCoV-19-Spain-AR-IBV-2423-2020	EPI-ISL-468859
hCoV-19-Spain-PV-IBV-2077-2020	EPI-ISL-468872
hCoV-19-Spain-PV-IBV-2130-2020	EPI-ISL-468878
hCoV-19-Spain-RI-IBV-99010924-2020	EPI-ISL-732763
hCoV-19-Spain-NC-CHN-01000307-2020	EPI-ISL-1382909
hCoV-19-Spain-NC-CHN-01000239-2020	EPI-ISL-1382910
hCoV-19-Spain-NC-CHN-01000243-2020	EPI-ISL-1382911
hCoV-19-Spain-NC-CHN-01000148-2020	EPI-ISL-1382912
hCoV-19-Spain-NC-CHN-01000165-2020	EPI-ISL-1382914
hCoV-19-Spain-NC-CHN-01000072-2020	EPI-ISL-1382915
hCoV-19-Spain-NC-CHN-01000162-2020	EPI-ISL-1382927
hCoV-19-Spain-NC-CHN-01000245-2020	EPI-ISL-1382943
hCoV-19-Spain-NC-CHN-01000253-2020	EPI-ISL-1382945
hCoV-19-Spain-NC-CHN-01000254-2020	EPI-ISL-1382946
hCoV-19-Spain-NC-CHN-01000256-2020	EPI-ISL-1382947
hCoV-19-Spain-NC-CHN-01000258-2020	EPI-ISL-1382949
hCoV-19-Spain-NC-CHN-01000260-2020	EPI-ISL-1382950
hCoV-19-Spain-NC-CHN-01000262-2020	EPI-ISL-1382952
hCoV-19-Spain-NC-CHN-01000263-2020	EPI-ISL-1382953
hCoV-19-Spain-NC-CHN-01000272-2020	EPI-ISL-1382961
hCoV-19-Spain-NC-CHN-01000274-2020	EPI-ISL-1382963
hCoV-19-Spain-NC-CHN-01000276-2020	EPI-ISL-1382965
hCoV-19-Spain-NC-CHN-01000277-2020	EPI-ISL-1382966
hCoV-19-Spain-NC-CHN-01000290-2020	EPI-ISL-1382977
hCoV-19-Spain-VC-FISABIO-222-2020	EPI-ISL-436317
hCoV-19-Spain-VC-FISABIO-230-2020	EPI-ISL-436325
hCoV-19-Spain-VC-FISABIO-233-2020	EPI-ISL-436328
hCoV-19-Spain-VC-FISABIO-235-2020	EPI-ISL-436330
hCoV-19-Spain-VC-FISABIO-241-2020	EPI-ISL-436336
hCoV-19-Spain-VC-FISABIO-261-2020	EPI-ISL-436356
hCoV-19-Spain-VC-FISABIO-271-2020	EPI-ISL-436366
hCoV-19-Spain-VC-FISABIO-273-2020	EPI-ISL-436368
hCoV-19-Spain-VC-FISABIO-274-2020	EPI-ISL-436369
hCoV-19-Spain-VC-FISABIO-276-2020	EPI-ISL-436371
hCoV-19-Spain-VC-FISABIO-281-2020	EPI-ISL-436376
hCoV-19-Spain-VC-FISABIO-291-2020	EPI-ISL-436386
hCoV-19-Spain-VC-FISABIO-296-2020	EPI-ISL-436391
hCoV-19-Spain-VC-FISABIO-302-2020	EPI-ISL-436397
hCoV-19-Spain-CM-ISCIII-202018188-2020	EPI-ISL-539526
hCoV-19-Spain-CM-ISCIII-202018234-2020	EPI-ISL-539527
hCoV-19-Spain-CM-ISCIII-202018271-2020	EPI-ISL-539528
hCoV-19-Spain-VC-FISABIO-215-2020	EPI-ISL-436310
hCoV-19-Spain-VC-FISABIO-221-2020	EPI-ISL-436316
hCoV-19-Spain-AR-IBV-2461-2020	EPI-ISL-468781
hCoV-19-Spain-AR-IBV-2409-2020	EPI-ISL-468782
hCoV-19-Spain-CL-ISCIII-202468-2020	EPI-ISL-539524
hCoV-19-Spain-CL-ISCIII-204996-2020	EPI-ISL-539525
hCoV-19-Spain-CM-ISCIII-202018315-2020	EPI-ISL-539529
hCoV-19-Spain-CM-ISCIII-202018318-2020	EPI-ISL-539530
hCoV-19-Spain-CT-ISCIII-209811-2020	EPI-ISL-539552
hCoV-19-Spain-CT-ISCIII-209814-2020	EPI-ISL-539553

hCoV-19-Spain-CT-ISCIII-209815-2020	EPI-ISL-539554
hCoV-19-Spain-CT-HUVH-49385-2020	EPI-ISL-819295
hCoV-19-Spain-CT-HUVH-53217-2020	EPI-ISL-819297
hCoV-19-Spain-CT-HUVH-VH7924-2020	EPI-ISL-450337
hCoV-19-Spain-MD-IBV-003705-2020	EPI-ISL-467184
hCoV-19-Spain-MD-IBV-003687-2020	EPI-ISL-467186
hCoV-19-Spain-MD-IBV-003669-2020	EPI-ISL-467188
hCoV-19-Spain-MD-IBV-003663-2020	EPI-ISL-467189
hCoV-19-Spain-MD-IBV-003703-2020	EPI-ISL-467191
hCoV-19-Spain-MD-IBV-003708-2020	EPI-ISL-467199
hCoV-19-Spain-AN-IBV-98001736-2020	EPI-ISL-538141
hCoV-19-Spain-AN-IBV-98001776-2020	EPI-ISL-538151
hCoV-19-Spain-AN-IBV-98001778-2020	EPI-ISL-538152
hCoV-19-Spain-AN-IBV-98001786-2020	EPI-ISL-538156
hCoV-19-Spain-AN-IBV-98001806-2020	EPI-ISL-538162
hCoV-19-Spain-AN-IBV-98001807-2020	EPI-ISL-538163
hCoV-19-Spain-VC-IBV-98004362-2020	EPI-ISL-796071
hCoV-19-Spain-VC-IBV-98004373-2020	EPI-ISL-796076
hCoV-19-Spain-VC-IBV-98004376-2020	EPI-ISL-796077
hCoV-19-Spain-VC-IBV-98004505-2020	EPI-ISL-796094
hCoV-19-Spain-VC-IBV-98004506-2020	EPI-ISL-796095
hCoV-19-Spain-VC-IBV-98004510-2020	EPI-ISL-796096
hCoV-19-Spain-VC-IBV-98004511-2020	EPI-ISL-796097
hCoV-19-Spain-PV-IBV-001477-2020	EPI-ISL-467128
hCoV-19-Spain-PV-IBV-001555-2020	EPI-ISL-467138
hCoV-19-Spain-PV-IBV-001455-2020	EPI-ISL-467145
hCoV-19-Spain-PV-IBV-001445-2020	EPI-ISL-467148
hCoV-19-Spain-PV-IBV-001561-2020	EPI-ISL-467151
hCoV-19-Spain-PV-IBV-001500-2020	EPI-ISL-467152
hCoV-19-Spain-PV-IBV-001525-2020	EPI-ISL-467167
hCoV-19-Spain-AN-IBV-002017-2020	EPI-ISL-509628
hCoV-19-Spain-AN-IBV-002020-2020	EPI-ISL-509630
hCoV-19-Spain-PV-IBV-004181-2020	EPI-ISL-510468
hCoV-19-Spain-PV-IBV-004182-2020	EPI-ISL-510469
hCoV-19-Spain-PV-IBV-004183-2020	EPI-ISL-510470
hCoV-19-Spain-PV-IBV-004186-2020	EPI-ISL-510473
hCoV-19-Spain-PV-IBV-004200-2020	EPI-ISL-510485
hCoV-19-Spain-PV-IBV-004204-2020	EPI-ISL-510489
hCoV-19-Spain-AN-IBV-98001631-2020	EPI-ISL-538119
hCoV-19-Spain-AN-IBV-98001689-2020	EPI-ISL-538128
hCoV-19-Spain-AN-IBV-98001690-2020	EPI-ISL-538129
hCoV-19-Spain-VC-IBV-98005513-2020	EPI-ISL-796127
hCoV-19-Spain-VC-IBV-98005519-2020	EPI-ISL-796128
hCoV-19-Spain-VC-IBV-98005520-2020	EPI-ISL-796129
hCoV-19-Spain-VC-IBV-98006943-2020	EPI-ISL-796136
hCoV-19-Spain-VC-IBV-98006950-2020	EPI-ISL-796139
hCoV-19-Spain-VC-IBV-98006954-2020	EPI-ISL-796141
hCoV-19-Spain-VC-IBV-98006956-2020	EPI-ISL-796142
hCoV-19-Spain-VC-IBV-98006961-2020	EPI-ISL-796143
hCoV-19-Spain-VC-IBV-98006964-2020	EPI-ISL-796145
hCoV-19-Spain-VC-IBV-98006966-2020	EPI-ISL-796147
hCoV-19-Spain-VC-IBV-98006972-2020	EPI-ISL-796149
hCoV-19-Spain-VC-IBV-98006974-2020	EPI-ISL-796150
hCoV-19-Spain-VC-IBV-98006978-2020	EPI-ISL-796153
hCoV-19-Spain-VC-IBV-98006981-2020	EPI-ISL-796156
hCoV-19-Spain-MD-IBV-99013150-2020	EPI-ISL-831039
hCoV-19-Spain-CT-IrsiCaixa-sp50-2020	EPI-ISL-462447
hCoV-19-Spain-CT-IrsiCaixa-sp142-2020	EPI-ISL-462448
hCoV-19-Spain-CT-IrsiCaixa-015D0-2020	EPI-ISL-462449
hCoV-19-Spain-PV-IBV-004287-2020	EPI-ISL-510403
hCoV-19-Spain-PV-IBV-004291-2020	EPI-ISL-510407
hCoV-19-Spain-PV-IBV-004304-2020	EPI-ISL-510419
hCoV-19-Spain-PV-IBV-004208-2020	EPI-ISL-510493
hCoV-19-Spain-PV-IBV-004210-2020	EPI-ISL-510495
hCoV-19-Spain-VC-IBV-98004540-2020	EPI-ISL-796110
hCoV-19-Spain-VC-IBV-98004548-2020	EPI-ISL-796111
hCoV-19-Spain-VC-IBV-98004556-2020	EPI-ISL-796114
hCoV-19-Spain-VC-IBV-98004572-2020	EPI-ISL-796117

hCoV-19-Spain-VC-IBV-98004583-2020	EPI-ISL-796119
hCoV-19-Spain-VC-IBV-98004586-2020	EPI-ISL-796122
hCoV-19-Spain-MD-IBV-99013151-2020	EPI-ISL-831040
hCoV-19-Spain-MD-IBV-99013153-2020	EPI-ISL-831042
hCoV-19-Spain-MD-IBV-99013156-2020	EPI-ISL-831043
hCoV-19-Spain-MD-IBV-99013158-2020	EPI-ISL-831045
hCoV-19-Spain-MD-IBV-99013159-2020	EPI-ISL-831046
hCoV-19-Spain-MD-IBV-99013160-2020	EPI-ISL-831047
hCoV-19-Spain-MD-IBV-99013163-2020	EPI-ISL-831050
hCoV-19-Spain-MD-IBV-99013164-2020	EPI-ISL-831051
hCoV-19-Spain-MD-IBV-99013166-2020	EPI-ISL-831053
hCoV-19-Spain-MD-IBV-99013168-2020	EPI-ISL-831054
hCoV-19-Spain-MD-IBV-99013169-2020	EPI-ISL-831055
hCoV-19-Spain-MD-IBV-99013170-2020	EPI-ISL-831056
hCoV-19-Spain-MD-IBV-99013171-2020	EPI-ISL-831057
hCoV-19-Spain-MD-IBV-99013172-2020	EPI-ISL-831058
hCoV-19-Spain-MD-IBV-99013173-2020	EPI-ISL-831059
hCoV-19-Spain-MD-IBV-99013174-2020	EPI-ISL-831060
hCoV-19-Spain-MD-IBV-99013175-2020	EPI-ISL-831061
hCoV-19-Spain-MD-IBV-99013176-2020	EPI-ISL-831062
hCoV-19-Spain-MD-IBV-99013177-2020	EPI-ISL-831063
hCoV-19-Spain-MD-IBV-99013178-2020	EPI-ISL-831064
hCoV-19-Spain-MD-IBV-99013180-2020	EPI-ISL-831066
hCoV-19-Spain-MD-IBV-99013182-2020	EPI-ISL-831068
hCoV-19-Spain-MD-IBV-99013184-2020	EPI-ISL-831070
hCoV-19-Spain-MD-IBV-99013185-2020	EPI-ISL-831071
hCoV-19-Spain-MD-IBV-99013186-2020	EPI-ISL-831072
hCoV-19-Spain-MD-IBV-99013188-2020	EPI-ISL-831073
hCoV-19-Spain-MD-IBV-99013190-2020	EPI-ISL-831075
hCoV-19-Spain-MD-IBV-99013191-2020	EPI-ISL-831076
hCoV-19-Spain-MD-IBV-99013193-2020	EPI-ISL-831078
hCoV-19-Spain-MD-IBV-99013194-2020	EPI-ISL-831079
hCoV-19-Spain-MD-IBV-99013195-2020	EPI-ISL-831080
hCoV-19-Spain-MD-IBV-99013196-2020	EPI-ISL-831081
hCoV-19-Spain-MD-IBV-003660-2020	EPI-ISL-467222
hCoV-19-Spain-MD-IBV-003649-2020	EPI-ISL-467226
hCoV-19-Spain-MD-IBV-003648-2020	EPI-ISL-467228
hCoV-19-Spain-MD-IBV-003677-2020	EPI-ISL-467230
hCoV-19-Spain-MD-IBV-003702-2020	EPI-ISL-467235
hCoV-19-Spain-MD-IBV-003716-2020	EPI-ISL-467238
hCoV-19-Spain-MD-IBV-003717-2020	EPI-ISL-467241
hCoV-19-Spain-GA-IBV-002713-2020	EPI-ISL-467293
hCoV-19-Spain-GA-IBV-002723-2020	EPI-ISL-467295
hCoV-19-Spain-GA-IBV-002719-2020	EPI-ISL-467297
hCoV-19-Spain-PV-HUD-70063452-2020	EPI-ISL-475719
hCoV-19-Spain-PV-IBV-004219-2020	EPI-ISL-510503
hCoV-19-Spain-PV-IBV-004222-2020	EPI-ISL-510505
hCoV-19-Spain-MD-H12-37-2020	EPI-ISL-529972
hCoV-19-Spain-MD-H12-38-2020	EPI-ISL-529973
hCoV-19-Spain-MD-H12-39-2020	EPI-ISL-529974
hCoV-19-Spain-MD-H12-40-2020	EPI-ISL-529975
hCoV-19-Spain-MD-H12-41-2020	EPI-ISL-529976
hCoV-19-Spain-MD-H12-42-2020	EPI-ISL-529977
hCoV-19-Spain-MD-H12-43-2020	EPI-ISL-529978
hCoV-19-Spain-MD-H12-47-2020	EPI-ISL-529980
hCoV-19-Spain-MD-H12-48-2020	EPI-ISL-529981
hCoV-19-Spain-MD-H12-49-2020	EPI-ISL-529982
hCoV-19-Spain-MD-H12-52-2020	EPI-ISL-529984
hCoV-19-Spain-MD-H12-53-2020	EPI-ISL-529985
hCoV-19-Spain-MD-H12-54-2020	EPI-ISL-529986
hCoV-19-Spain-MD-H12-55-2020	EPI-ISL-529987
hCoV-19-Spain-MD-H12-56-2020	EPI-ISL-529988
hCoV-19-Spain-MD-H12-57-2020	EPI-ISL-529989
hCoV-19-Spain-MD-H12-58-2020	EPI-ISL-529990
hCoV-19-Spain-MD-H12-59-2020	EPI-ISL-529991
hCoV-19-Spain-MD-H12-65-2020	EPI-ISL-529996
hCoV-19-Spain-MD-H12-66-2020	EPI-ISL-529997
hCoV-19-Spain-MD-H12-67-2020	EPI-ISL-529998

hCoV-19-Spain-MD-H12-68-2020	EPI-ISL-529999
hCoV-19-Spain-VC-IBV-98003099-2020	EPI-ISL-538035
hCoV-19-Spain-VC-IBV-98003100-2020	EPI-ISL-538036
hCoV-19-Spain-VC-IBV-98003101-2020	EPI-ISL-538037
hCoV-19-Spain-VC-IBV-98003102-2020	EPI-ISL-538038
hCoV-19-Spain-VC-IBV-98003107-2020	EPI-ISL-538043
hCoV-19-Spain-VC-IBV-98003109-2020	EPI-ISL-538045
hCoV-19-Spain-VC-IBV-98003114-2020	EPI-ISL-538050
hCoV-19-Spain-VC-IBV-98003115-2020	EPI-ISL-538051
hCoV-19-Spain-VC-IBV-98003116-2020	EPI-ISL-538052
hCoV-19-Spain-VC-IBV-98003118-2020	EPI-ISL-538054
hCoV-19-Spain-CT-IBV-97013493-2020	EPI-ISL-1008885
hCoV-19-Spain-MD-IBV-003697-2020	EPI-ISL-467207
hCoV-19-Spain-MD-IBV-003668-2020	EPI-ISL-467209
hCoV-19-Spain-MD-IBV-003665-2020	EPI-ISL-467215
hCoV-19-Spain-MD-IBV-003678-2020	EPI-ISL-467217
hCoV-19-Spain-MD-IBV-003670-2020	EPI-ISL-467218
hCoV-19-Spain-MD-IBV-003664-2020	EPI-ISL-467254
hCoV-19-Spain-MD-IBV-003646-2020	EPI-ISL-467255
hCoV-19-Spain-MD-IBV-003698-2020	EPI-ISL-467256
hCoV-19-Spain-MD-IBV-003696-2020	EPI-ISL-467257
hCoV-19-Spain-MD-IBV-003700-2020	EPI-ISL-467259
hCoV-19-Spain-MD-IBV-003676-2020	EPI-ISL-467260
hCoV-19-Spain-MD-IBV-003693-2020	EPI-ISL-467261
hCoV-19-Spain-GA-IBV-002714-2020	EPI-ISL-467263
hCoV-19-Spain-GA-IBV-002756-2020	EPI-ISL-467267
hCoV-19-Spain-GA-IBV-002710-2020	EPI-ISL-467278
hCoV-19-Spain-GA-IBV-002718-2020	EPI-ISL-467279
hCoV-19-Spain-GA-IBV-002733-2020	EPI-ISL-467289
hCoV-19-Spain-CB-IBV-98007086-2020	EPI-ISL-582109
hCoV-19-Spain-CT-HUVH-51107-2020	EPI-ISL-819301
hCoV-19-Spain-CT-HUVH-51925-2020	EPI-ISL-819302
hCoV-19-Spain-CT-HUVH-13372-2020	EPI-ISL-819303
hCoV-19-Spain-CT-HUVH-52036-2020	EPI-ISL-819304
hCoV-19-Spain-CT-HUVH-14601-2020	EPI-ISL-819305
hCoV-19-Spain-CT-HUVH-14643-2020	EPI-ISL-819306
hCoV-19-Spain-CT-HUVH-16634-2020	EPI-ISL-819307
hCoV-19-Spain-CT-HUVH-16830-2020	EPI-ISL-819308
hCoV-19-Spain-CT-HUVH-16886-2020	EPI-ISL-819309
hCoV-19-Spain-CT-HUVH-52489-2020	EPI-ISL-819310
hCoV-19-Spain-CT-HUVH-18228-2020	EPI-ISL-819311
hCoV-19-Spain-CT-HUVH-21918-2020	EPI-ISL-819312
hCoV-19-Spain-CT-HUVH-22661-2020	EPI-ISL-819313
hCoV-19-Spain-CT-HUVH-57694-2020	EPI-ISL-819314
hCoV-19-Spain-CT-HUVH-23216-2020	EPI-ISL-819315
hCoV-19-Spain-CT-HUVH-58300-2020	EPI-ISL-819316
hCoV-19-Spain-CT-HUVH-58833-2020	EPI-ISL-819317
hCoV-19-Spain-CT-HUVH-26864-2020	EPI-ISL-819318
hCoV-19-Spain-CT-HUVH-60715-2020	EPI-ISL-819319
hCoV-19-Spain-CT-HUVH-29000-2020	EPI-ISL-819322
hCoV-19-Spain-CT-HUVH-32192-2020	EPI-ISL-819323
hCoV-19-Spain-CT-HUVH-33417-2020	EPI-ISL-819324
hCoV-19-Spain-CT-HUVH-36647-2020	EPI-ISL-819327
hCoV-19-Spain-CT-HUVH-37867-2020	EPI-ISL-819328
hCoV-19-Spain-CT-HUVH-39817-2020	EPI-ISL-819330
hCoV-19-Spain-CT-HUVH-41174-2020	EPI-ISL-819331
hCoV-19-Spain-CT-HUVH-41219-2020	EPI-ISL-819332
hCoV-19-Spain-CT-HUVH-42151-2020	EPI-ISL-819333
hCoV-19-Spain-CT-HUVH-43512-2020	EPI-ISL-819334
hCoV-19-Spain-CT-HUVH-39637-2020	EPI-ISL-819335
hCoV-19-Spain-CT-HUVH-VH1133-2020	EPI-ISL-418860
hCoV-19-Spain-CM-IBV-003758-2020	EPI-ISL-474933
hCoV-19-Spain-MC-IBV-002894-2020	EPI-ISL-474934
hCoV-19-Spain-AN-IBV-003033-2020	EPI-ISL-474935
hCoV-19-Spain-AN-IBV-002885-2020	EPI-ISL-474936
hCoV-19-Spain-AN-IBV-002918-2020	EPI-ISL-474939
hCoV-19-Spain-CT-IrsiCaixa-Own160320-2020	EPI-ISL-483059

hCoV-19-Spain-MD-H12-69-2020	EPI-ISL-530000
hCoV-19-Spain-MD-H12-70-2020	EPI-ISL-530001
hCoV-19-Spain-MD-H12-71-2020	EPI-ISL-530002
hCoV-19-Spain-MD-H12-72-2020	EPI-ISL-530003
hCoV-19-Spain-RI-IBV-99005892-2020	EPI-ISL-537434
hCoV-19-Spain-RI-IBV-99005908-2020	EPI-ISL-537446
hCoV-19-Spain-AN-IBV-002983-2020	EPI-ISL-474908
hCoV-19-Spain-CN-IBV-002596-2020	EPI-ISL-474912
hCoV-19-Spain-CN-IBV-002599-2020	EPI-ISL-474915
hCoV-19-Spain-CT-IBV-003729-2020	EPI-ISL-480981
hCoV-19-Spain-PV-IBV-003238-2020	EPI-ISL-480983
hCoV-19-Spain-PV-IBV-003230-2020	EPI-ISL-480988
hCoV-19-Spain-VC-FISABIO-522-2020	EPI-ISL-447473
hCoV-19-Spain-VC-FISABIO-523-2020	EPI-ISL-447474
hCoV-19-Spain-VC-FISABIO-529-2020	EPI-ISL-447478
hCoV-19-Spain-VC-FISABIO-540-2020	EPI-ISL-447485
hCoV-19-Spain-VC-FISABIO-551-2020	EPI-ISL-447493
hCoV-19-Spain-VC-FISABIO-565-2020	EPI-ISL-447497
hCoV-19-Spain-CT-HUVH-VH8002-2020	EPI-ISL-458130
hCoV-19-Spain-CT-HUVH-VH9461-2020	EPI-ISL-458131
hCoV-19-Spain-CT-HUVH-VH9758-2020	EPI-ISL-458132
hCoV-19-Spain-CT-HUVH-VH7458-2020	EPI-ISL-444974
hCoV-19-Spain-CT-HUVH-VH1407-2020	EPI-ISL-444975
hCoV-19-Spain-CT-HUVH-VH7773-2020	EPI-ISL-444976
hCoV-19-Spain-CT-HUVH-VH1438-2020	EPI-ISL-444977
hCoV-19-Spain-CT-HUVH-VH4141-2020	EPI-ISL-444978
hCoV-19-Spain-CT-HUVH-VH4488-2020	EPI-ISL-444979
hCoV-19-Spain-CT-HUVH-VH4964-2020	EPI-ISL-444980
hCoV-19-Spain-CT-HUVH-VH5081-2020	EPI-ISL-444981
hCoV-19-Spain-CT-HUVH-VH5325-2020	EPI-ISL-444982
hCoV-19-Spain-CT-HUVH-VH5994-2020	EPI-ISL-444983
hCoV-19-Spain-CT-HUVH-VH6552-2020	EPI-ISL-444984
hCoV-19-Spain-CT-HUVH-VH6837-2020	EPI-ISL-444985
hCoV-19-Spain-CT-HUVH-VH8938-2020	EPI-ISL-444986
hCoV-19-Spain-CT-HUVH-VH9434-2020	EPI-ISL-444987
hCoV-19-Spain-CT-HUVH-VH0851-2020	EPI-ISL-444988
hCoV-19-Spain-CT-HUVH-VH1484-2020	EPI-ISL-444989
hCoV-19-Spain-PV-IBV-2096-2020	EPI-ISL-468933
hCoV-19-Spain-PV-IBV-2129-2020	EPI-ISL-468940
hCoV-19-Spain-PV-IBV-2084-2020	EPI-ISL-468949
hCoV-19-Spain-IB-IBV-2591-2020	EPI-ISL-468966
hCoV-19-Spain-IB-IBV-2542-2020	EPI-ISL-468975
hCoV-19-Spain-IB-IBV-2506-2020	EPI-ISL-468976
hCoV-19-Spain-CT-HUVH-VH6372-2020	EPI-ISL-444971
hCoV-19-Spain-CT-HUVH-VH6819-2020	EPI-ISL-444972
hCoV-19-Spain-CT-HUVH-VH7280-2020	EPI-ISL-444973
hCoV-19-Spain-PV-IBV-2081-2020	EPI-ISL-468920
hCoV-19-Spain-PV-IBV-2128-2020	EPI-ISL-468926
hCoV-19-Spain-VC-FISABIO-316-2020	EPI-ISL-436411
hCoV-19-Spain-VC-FISABIO-661-2020	EPI-ISL-455727
hCoV-19-Spain-CM-IBV-003760-2020	EPI-ISL-474803
hCoV-19-Spain-CM-IBV-003777-2020	EPI-ISL-474810
hCoV-19-Spain-CM-IBV-003786-2020	EPI-ISL-474815
hCoV-19-Spain-CM-IBV-003792-2020	EPI-ISL-474818
hCoV-19-Spain-CM-IBV-003894-2020	EPI-ISL-474830
hCoV-19-Spain-AN-IBV-002998-2020	EPI-ISL-474836
hCoV-19-Spain-AN-IBV-002912-2020	EPI-ISL-474851
hCoV-19-Spain-AN-IBV-002974-2020	EPI-ISL-474856
hCoV-19-Spain-AN-IBV-002889-2020	EPI-ISL-474867
hCoV-19-Spain-AN-IBV-002908-2020	EPI-ISL-474869
hCoV-19-Spain-AN-IBV-002930-2020	EPI-ISL-474874
hCoV-19-Spain-AN-IBV-003007-2020	EPI-ISL-474889
hCoV-19-Spain-CT-IBV-005626-2020	EPI-ISL-508661
hCoV-19-Spain-CT-IBV-005634-2020	EPI-ISL-508667
hCoV-19-Spain-CT-IBV-005635-2020	EPI-ISL-508676
hCoV-19-Spain-AS-IBV-98006648-2020	EPI-ISL-654634
hCoV-19-Spain-AS-IBV-98006618-2020	EPI-ISL-654658
hCoV-19-Spain-AS-IBV-98006620-2020	EPI-ISL-654659

hCoV-19-Spain-AS-IBV-98006623-2020	EPI-ISL-654660
hCoV-19-Spain-AS-IBV-98006629-2020	EPI-ISL-654663
hCoV-19-Spain-AS-IBV-98006630-2020	EPI-ISL-654664
hCoV-19-Spain-AS-IBV-98006632-2020	EPI-ISL-654666
hCoV-19-Spain-AS-IBV-98006634-2020	EPI-ISL-654667
hCoV-19-Spain-AS-IBV-98006638-2020	EPI-ISL-654668
hCoV-19-Spain-AS-IBV-98006643-2020	EPI-ISL-654669
hCoV-19-Spain-AS-IBV-98006644-2020	EPI-ISL-654670
hCoV-19-Spain-AS-IBV-98006656-2020	EPI-ISL-654671
hCoV-19-Spain-AS-IBV-98006617-2020	EPI-ISL-654673
hCoV-19-Spain-AS-IBV-98006650-2020	EPI-ISL-654674
hCoV-19-Spain-AS-IBV-98006621-2020	EPI-ISL-654675
hCoV-19-Spain-AS-IBV-98006635-2020	EPI-ISL-654676
hCoV-19-Spain-AS-IBV-98006636-2020	EPI-ISL-654677
hCoV-19-Spain-AS-IBV-98006641-2020	EPI-ISL-654678
hCoV-19-Spain-AS-IBV-98006642-2020	EPI-ISL-654679
hCoV-19-Spain-AS-IBV-98006655-2020	EPI-ISL-654680
hCoV-19-Spain-AS-IBV-98006657-2020	EPI-ISL-654681
hCoV-19-Spain-AS-IBV-98006627-2020	EPI-ISL-654683
hCoV-19-Spain-MD-H12-20-2020	EPI-ISL-428684
hCoV-19-Spain-MD-H12-23-2020	EPI-ISL-428686
hCoV-19-Spain-MD-H12-24-2020	EPI-ISL-428687
hCoV-19-Spain-MD-H12-25-2020	EPI-ISL-428688
hCoV-19-Spain-MD-H12-26-2020	EPI-ISL-428689
hCoV-19-Spain-MD-H12-27-2020	EPI-ISL-428690
hCoV-19-Spain-AN-IBV-002041-2020	EPI-ISL-452385
hCoV-19-Spain-RI-IBV-98002323-2020	EPI-ISL-537842
hCoV-19-Spain-AS-HUCA-232025446-2020	EPI-ISL-710491
hCoV-19-Spain-AS-HUCA-232039084-2020	EPI-ISL-710492
hCoV-19-Spain-CL-ISCIII-206602-2020	EPI-ISL-862639
hCoV-19-Spain-CL-ISCIII-206201-2020	EPI-ISL-862640
hCoV-19-Spain-MD-H12-1804-2020	EPI-ISL-421174
hCoV-19-Spain-MD-H12-H12-1905-2020	EPI-ISL-421175
hCoV-19-Spain-MD-H12-2006-2020	EPI-ISL-421176
hCoV-19-Spain-MD-H12-H12-2107-2020	EPI-ISL-421177
hCoV-19-Spain-MD-H12-H12-2208-2020	EPI-ISL-421178
hCoV-19-Spain-MD-H12-2410-2020	EPI-ISL-421180
hCoV-19-Spain-CN-IBV-97006508-2020	EPI-ISL-537716
hCoV-19-Spain-NC-IBV-001416-2020	EPI-ISL-452485
hCoV-19-Spain-NC-IBV-001415-2020	EPI-ISL-452486
hCoV-19-Spain-NC-IBV-001406-2020	EPI-ISL-452495
hCoV-19-Spain-NC-IBV-001403-2020	EPI-ISL-452498
hCoV-19-Spain-RI-IBV-005987-2020	EPI-ISL-500421
hCoV-19-Spain-RI-IBV-006002-2020	EPI-ISL-500433
hCoV-19-Spain-CN-IBV-97006516-2020	EPI-ISL-537721
hCoV-19-Spain-CN-IBV-97006519-2020	EPI-ISL-537724
hCoV-19-Spain-MD-H12-2902-2020	EPI-ISL-428701
hCoV-19-Spain-MD-H12-3506-2020	EPI-ISL-428705
hCoV-19-Spain-MD-H12-3607-2020	EPI-ISL-428706
hCoV-19-Spain-MD-H12-3708-2020	EPI-ISL-428707
hCoV-19-Spain-MD-H12-3809-2020	EPI-ISL-428708
hCoV-19-Spain-MD-H12-3910-2020	EPI-ISL-428709
hCoV-19-Spain-MD-H12-4112-2020	EPI-ISL-428711
hCoV-19-Spain-CN-IBV-97006485-2020	EPI-ISL-537700
hCoV-19-Spain-GA-CHUVI-21322168-2020	EPI-ISL-2285148
hCoV-19-Spain-GA-CHUVI-21322296-2020	EPI-ISL-2285149
hCoV-19-Spain-GA-CHUVI-33196285-2020	EPI-ISL-2285152
hCoV-19-Spain-VC-FISABIO-576-2020	EPI-ISL-447506
hCoV-19-Spain-VC-FISABIO-582-2020	EPI-ISL-447512
hCoV-19-Spain-VC-FISABIO-583-2020	EPI-ISL-447513
hCoV-19-Spain-VC-FISABIO-584-2020	EPI-ISL-447514
hCoV-19-Spain-VC-FISABIO-585-2020	EPI-ISL-447515
hCoV-19-Spain-VC-FISABIO-594-2020	EPI-ISL-447517

hCoV-19-Spain-VC-FISABIO-604-2020	EPI-ISL-447523
hCoV-19-Spain-VC-FISABIO-613-2020	EPI-ISL-447531
hCoV-19-Spain-CT-HUVH-VH1449-2020	EPI-ISL-447532
hCoV-19-Spain-CT-HUVH-VH7663-2020	EPI-ISL-447533
hCoV-19-Spain-CT-IBV-99012650-2020	EPI-ISL-949240
hCoV-19-Spain-NC-CHN-01000018-2020	EPI-ISL-1383203
hCoV-19-Spain-NC-CHN-01000020-2020	EPI-ISL-1383205
hCoV-19-Spain-NC-CHN-01000025-2020	EPI-ISL-1383206
hCoV-19-Spain-NC-CHN-01000026-2020	EPI-ISL-1383207
hCoV-19-Spain-NC-CHN-01000041-2020	EPI-ISL-1383213
hCoV-19-Spain-NC-CHN-01000042-2020	EPI-ISL-1383214
hCoV-19-Spain-NC-CHN-01000059-2020	EPI-ISL-1383221
hCoV-19-Spain-NC-CHN-01000074-2020	EPI-ISL-1383224
hCoV-19-Spain-NC-CHN-01000075-2020	EPI-ISL-1383225
hCoV-19-Spain-NC-CHN-01000082-2020	EPI-ISL-1383227
hCoV-19-Spain-NC-CHN-01000083-2020	EPI-ISL-1383228
hCoV-19-Spain-MD-H12-73-2020	EPI-ISL-530004
hCoV-19-Spain-MD-H12-80-2020	EPI-ISL-530009
hCoV-19-Spain-MD-H12-81-2020	EPI-ISL-530010
hCoV-19-Spain-MD-H12-82-2020	EPI-ISL-530011
hCoV-19-Spain-MD-H12-83-2020	EPI-ISL-530012
hCoV-19-Spain-MD-H12-84-2020	EPI-ISL-530013
hCoV-19-Spain-MD-H12-85-2020	EPI-ISL-530014
hCoV-19-Spain-MD-H12-87-2020	EPI-ISL-530015
hCoV-19-Spain-MD-H12-90-2020	EPI-ISL-530017
hCoV-19-Spain-MD-H12-91-2020	EPI-ISL-530018
hCoV-19-Spain-MD-H12-93-2020	EPI-ISL-530019
hCoV-19-Spain-MD-H12-94-2020	EPI-ISL-530020
hCoV-19-Spain-MD-H12-95-2020	EPI-ISL-530021
hCoV-19-Spain-MD-H12-96-2020	EPI-ISL-530022
hCoV-19-Spain-MD-H12-98-2020	EPI-ISL-530024
hCoV-19-Spain-MD-HLP-100-1-2020	EPI-ISL-530025
hCoV-19-Spain-MD-HLP-101-2020	EPI-ISL-530027
hCoV-19-Spain-IB-IBV-2590-2020	EPI-ISL-469015
hCoV-19-Spain-MD-HLP-103-2020	EPI-ISL-530030
hCoV-19-Spain-MD-HLP-104-2-2020	EPI-ISL-530032
hCoV-19-Spain-MD-HLP-104-3-2020	EPI-ISL-530033
hCoV-19-Spain-MD-HLP-106-2-2020	EPI-ISL-530035
hCoV-19-Spain-MD-HLP-106-3-2020	EPI-ISL-530036
hCoV-19-Spain-MD-HLP-108-2020	EPI-ISL-530037
hCoV-19-Spain-MD-HLP-109-2020	EPI-ISL-530038
hCoV-19-Spain-MD-HLP-40-2020	EPI-ISL-530044
hCoV-19-Spain-MD-HLP-42-2020	EPI-ISL-530046
hCoV-19-Spain-MD-HLP-43-2020	EPI-ISL-530047
hCoV-19-Spain-MD-HLP-44-2020	EPI-ISL-530048
hCoV-19-Spain-MD-HLP-45-2020	EPI-ISL-530049
hCoV-19-Spain-MD-HLP-46-2020	EPI-ISL-530050
hCoV-19-Spain-MD-HLP-48-2020	EPI-ISL-530051
hCoV-19-Spain-MD-HLP-49-2020	EPI-ISL-530052
hCoV-19-Spain-MD-HLP-50-2020	EPI-ISL-530053
hCoV-19-Spain-MD-HLP-51-2020	EPI-ISL-530054
hCoV-19-Spain-MD-HLP-52-2020	EPI-ISL-530055
hCoV-19-Spain-MD-HLP-53-2020	EPI-ISL-530056
hCoV-19-Spain-MD-HLP-54-2020	EPI-ISL-530057
hCoV-19-Spain-MD-HLP-55-2020	EPI-ISL-530058
hCoV-19-Spain-MD-HLP-56-2020	EPI-ISL-530059
hCoV-19-Spain-MD-HLP-57-2020	EPI-ISL-530060
hCoV-19-Spain-MD-HLP-58-2020	EPI-ISL-530061
hCoV-19-Spain-MD-HLP-59-2020	EPI-ISL-530062
hCoV-19-Spain-MD-HLP-60-2020	EPI-ISL-530063
hCoV-19-Spain-MD-HLP-61-2020	EPI-ISL-530064
hCoV-19-Spain-MD-HLP-62-2020	EPI-ISL-530065
hCoV-19-Spain-MD-HLP-63-2020	EPI-ISL-530066
hCoV-19-Spain-MD-HLP-64-2020	EPI-ISL-530067
hCoV-19-Spain-MD-HLP-74-2020	EPI-ISL-530073
hCoV-19-Spain-MD-HLP-85-2020	EPI-ISL-530082
hCoV-19-Spain-MD-HLP-86-2020	EPI-ISL-530083
hCoV-19-Spain-MD-HLP-87-2020	EPI-ISL-530084

hCoV-19-Spain-MD-HLP-88-2020	EPI-ISL-530085
hCoV-19-Spain-NC-CHN-01000183-2020	EPI-ISL-1383122
hCoV-19-Spain-NC-CHN-01000185-2020	EPI-ISL-1383124
hCoV-19-Spain-NC-CHN-01000187-2020	EPI-ISL-1383126
hCoV-19-Spain-NC-CHN-01000188-2020	EPI-ISL-1383127
hCoV-19-Spain-NC-CHN-01000189-2020	EPI-ISL-1383128
hCoV-19-Spain-NC-CHN-01000007-2020	EPI-ISL-1383129
hCoV-19-Spain-NC-CHN-01000015-2020	EPI-ISL-1383131
hCoV-19-Spain-NC-CHN-01000023-2020	EPI-ISL-1383133
hCoV-19-Spain-NC-CHN-01000024-2020	EPI-ISL-1383134
hCoV-19-Spain-NC-CHN-01000039-2020	EPI-ISL-1383138
hCoV-19-Spain-NC-CHN-01000040-2020	EPI-ISL-1383139
hCoV-19-Spain-NC-CHN-01000046-2020	EPI-ISL-1383140
hCoV-19-Spain-NC-CHN-01000048-2020	EPI-ISL-1383142
hCoV-19-Spain-NC-CHN-01000054-2020	EPI-ISL-1383143
hCoV-19-Spain-NC-CHN-01000055-2020	EPI-ISL-1383144
hCoV-19-Spain-NC-CHN-01000056-2020	EPI-ISL-1383145
hCoV-19-Spain-NC-CHN-01000062-2020	EPI-ISL-1383146
hCoV-19-Spain-NC-CHN-01000078-2020	EPI-ISL-1383150
hCoV-19-Spain-NC-CHN-01000080-2020	EPI-ISL-1383151
hCoV-19-Spain-NC-CHN-01000087-2020	EPI-ISL-1383153
hCoV-19-Spain-NC-CHN-01000088-2020	EPI-ISL-1383154
hCoV-19-Spain-NC-CHN-01000094-2020	EPI-ISL-1383155
hCoV-19-Spain-NC-CHN-01000096-2020	EPI-ISL-1383157
hCoV-19-Spain-NC-CHN-01000099-2020	EPI-ISL-1383160
hCoV-19-Spain-NC-CHN-01000101-2020	EPI-ISL-1383162
hCoV-19-Spain-NC-CHN-01000102-2020	EPI-ISL-1383163
hCoV-19-Spain-NC-CHN-01000103-2020	EPI-ISL-1383164
hCoV-19-Spain-NC-CHN-01000104-2020	EPI-ISL-1383165
hCoV-19-Spain-NC-CHN-01000105-2020	EPI-ISL-1383166
hCoV-19-Spain-NC-CHN-01000106-2020	EPI-ISL-1383167
hCoV-19-Spain-NC-CHN-01000107-2020	EPI-ISL-1383168
hCoV-19-Spain-NC-CHN-01000110-2020	EPI-ISL-1383171
hCoV-19-Spain-NC-CHN-01000113-2020	EPI-ISL-1383172
hCoV-19-Spain-NC-CHN-01000125-2020	EPI-ISL-1383175
hCoV-19-Spain-NC-CHN-01000005-2020	EPI-ISL-1383176
hCoV-19-Spain-NC-CHN-01000006-2020	EPI-ISL-1383177
hCoV-19-Spain-NC-CHN-01000014-2020	EPI-ISL-1383179
hCoV-19-Spain-NC-CHN-01000022-2020	EPI-ISL-1383181
hCoV-19-Spain-NC-CHN-01000029-2020	EPI-ISL-1383182
hCoV-19-Spain-NC-CHN-01000045-2020	EPI-ISL-1383184
hCoV-19-Spain-NC-CHN-01000060-2020	EPI-ISL-1383186
hCoV-19-Spain-NC-CHN-01000061-2020	EPI-ISL-1383187
hCoV-19-Spain-NC-CHN-01000076-2020	EPI-ISL-1383189
hCoV-19-Spain-NC-CHN-01000077-2020	EPI-ISL-1383190
hCoV-19-Spain-NC-CHN-01000085-2020	EPI-ISL-1383192
hCoV-19-Spain-NC-CHN-01000092-2020	EPI-ISL-1383193
hCoV-19-Spain-NC-CHN-01000093-2020	EPI-ISL-1383194
hCoV-19-Spain-NC-CHN-01000002-2020	EPI-ISL-1383195
hCoV-19-Spain-NC-CHN-01000003-2020	EPI-ISL-1383196
hCoV-19-Spain-NC-CHN-01000004-2020	EPI-ISL-1383197
hCoV-19-Spain-NC-CHN-01000009-2020	EPI-ISL-1383198
hCoV-19-Spain-NC-CHN-01000010-2020	EPI-ISL-1383199
hCoV-19-Spain-AR-IBV-005072-2020	EPI-ISL-510359
hCoV-19-Spain-AR-IBV-005074-2020	EPI-ISL-510360
hCoV-19-Spain-AR-IBV-005075-2020	EPI-ISL-510361
hCoV-19-Spain-MD-HLP-R51-2020	EPI-ISL-530101
hCoV-19-Spain-MD-HLP-R59-2020	EPI-ISL-530105
hCoV-19-Spain-MD-HLP-R61-2020	EPI-ISL-530107
hCoV-19-Spain-MD-HLP-R62-2020	EPI-ISL-530108
hCoV-19-Spain-MD-HLP-R64-2020	EPI-ISL-530110
hCoV-19-Spain-MD-HLP-R67-2020	EPI-ISL-530112
hCoV-19-Spain-MD-HLP-105-4-2020	EPI-ISL-534333
hCoV-19-Spain-MD-HGUGM-559336-2020	EPI-ISL-777902
hCoV-19-Spain-NC-CHN-01000157-2020	EPI-ISL-1383102
hCoV-19-Spain-NC-CHN-01000158-2020	EPI-ISL-1383103
hCoV-19-Spain-NC-CHN-01000160-2020	EPI-ISL-1383104

hCoV-19-Spain-NC-CHN-01000161-2020	EPI-ISL-1383105
hCoV-19-Spain-NC-CHN-01000164-2020	EPI-ISL-1383107
hCoV-19-Spain-NC-CHN-01000166-2020	EPI-ISL-1383108
hCoV-19-Spain-NC-CHN-01000167-2020	EPI-ISL-1383109
hCoV-19-Spain-NC-CHN-01000168-2020	EPI-ISL-1383110
hCoV-19-Spain-NC-CHN-01000169-2020	EPI-ISL-1383111
hCoV-19-Spain-NC-CHN-01000174-2020	EPI-ISL-1383114
hCoV-19-Spain-NC-CHN-01000176-2020	EPI-ISL-1383116
hCoV-19-Spain-VC-FISABIO-63-2020	EPI-ISL-425187
hCoV-19-Spain-VC-FISABIO-64-2020	EPI-ISL-425188
hCoV-19-Spain-VC-FISABIO-69-2020	EPI-ISL-425193
hCoV-19-Spain-VC-FISABIO-75-2020	EPI-ISL-425199
hCoV-19-Spain-GA-IBV-004062-2020	EPI-ISL-510300
hCoV-19-Spain-GA-IBV-004063-2020	EPI-ISL-510301
hCoV-19-Spain-GA-IBV-004064-2020	EPI-ISL-510302
hCoV-19-Spain-AR-IBV-005112-2020	EPI-ISL-510382
hCoV-19-Spain-MD-IBV-97010079-2020	EPI-ISL-779762
hCoV-19-Spain-MD-IBV-97010081-2020	EPI-ISL-779764
hCoV-19-Spain-MD-IBV-97010085-2020	EPI-ISL-779765
hCoV-19-Spain-MD-IBV-97010087-2020	EPI-ISL-779766
hCoV-19-Spain-NC-CHN-01000329-2020	EPI-ISL-1383014
hCoV-19-Spain-NC-CHN-01000190-2020	EPI-ISL-1383015
hCoV-19-Spain-NC-CHN-01000191-2020	EPI-ISL-1383016
hCoV-19-Spain-NC-CHN-01000192-2020	EPI-ISL-1383017
hCoV-19-Spain-NC-CHN-01000194-2020	EPI-ISL-1383019
hCoV-19-Spain-NC-CHN-01000196-2020	EPI-ISL-1383020
hCoV-19-Spain-NC-CHN-01000200-2020	EPI-ISL-1383023
hCoV-19-Spain-NC-CHN-01000201-2020	EPI-ISL-1383024
hCoV-19-Spain-NC-CHN-01000203-2020	EPI-ISL-1383026
hCoV-19-Spain-NC-CHN-01000204-2020	EPI-ISL-1383027
hCoV-19-Spain-NC-CHN-01000205-2020	EPI-ISL-1383028
hCoV-19-Spain-NC-CHN-01000206-2020	EPI-ISL-1383029
hCoV-19-Spain-NC-CHN-01000209-2020	EPI-ISL-1383032
hCoV-19-Spain-NC-CHN-01000210-2020	EPI-ISL-1383033
hCoV-19-Spain-NC-CHN-01000213-2020	EPI-ISL-1383036
hCoV-19-Spain-NC-CHN-01000219-2020	EPI-ISL-1383042
hCoV-19-Spain-NC-CHN-01000224-2020	EPI-ISL-1383047
hCoV-19-Spain-NC-CHN-01000226-2020	EPI-ISL-1383049
hCoV-19-Spain-NC-CHN-01000228-2020	EPI-ISL-1383051
hCoV-19-Spain-NC-CHN-01000230-2020	EPI-ISL-1383052
hCoV-19-Spain-NC-CHN-01000234-2020	EPI-ISL-1383056
hCoV-19-Spain-NC-CHN-01000236-2020	EPI-ISL-1383057
hCoV-19-Spain-NC-CHN-01000237-2020	EPI-ISL-1383058
hCoV-19-Spain-NC-CHN-01000238-2020	EPI-ISL-1383059
hCoV-19-Spain-NC-CHN-01000241-2020	EPI-ISL-1383061
hCoV-19-Spain-NC-CHN-01000244-2020	EPI-ISL-1383063
hCoV-19-Spain-NC-CHN-01000246-2020	EPI-ISL-1383064
hCoV-19-Spain-NC-CHN-01000249-2020	EPI-ISL-1383065
hCoV-19-Spain-NC-CHN-01000250-2020	EPI-ISL-1383066
hCoV-19-Spain-NC-CHN-01000255-2020	EPI-ISL-1383067
hCoV-19-Spain-NC-CHN-01000111-2020	EPI-ISL-1383068
hCoV-19-Spain-NC-CHN-01000112-2020	EPI-ISL-1383069
hCoV-19-Spain-NC-CHN-01000118-2020	EPI-ISL-1383072
hCoV-19-Spain-NC-CHN-01000119-2020	EPI-ISL-1383073
hCoV-19-Spain-NC-CHN-01000128-2020	EPI-ISL-1383080
hCoV-19-Spain-NC-CHN-01000133-2020	EPI-ISL-1383081
hCoV-19-Spain-NC-CHN-01000136-2020	EPI-ISL-1383084
hCoV-19-Spain-NC-CHN-01000137-2020	EPI-ISL-1383085
hCoV-19-Spain-NC-CHN-01000138-2020	EPI-ISL-1383086
hCoV-19-Spain-NC-CHN-01000140-2020	EPI-ISL-1383088
hCoV-19-Spain-NC-CHN-01000143-2020	EPI-ISL-1383089
hCoV-19-Spain-NC-CHN-01000144-2020	EPI-ISL-1383090
hCoV-19-Spain-NC-CHN-01000145-2020	EPI-ISL-1383091
hCoV-19-Spain-NC-CHN-01000146-2020	EPI-ISL-1383092
hCoV-19-Spain-NC-CHN-01000147-2020	EPI-ISL-1383093
hCoV-19-Spain-NC-CHN-01000149-2020	EPI-ISL-1383094
hCoV-19-Spain-NC-CHN-01000151-2020	EPI-ISL-1383096
hCoV-19-Spain-VC-IBV-98003441-2020	EPI-ISL-539251

hCoV-19-Spain-VC-IBV-98003443-2020	EPI-ISL-539252
hCoV-19-Spain-VC-IBV-98003445-2020	EPI-ISL-539254
hCoV-19-Spain-VC-IBV-98003446-2020	EPI-ISL-539255
hCoV-19-Spain-VC-IBV-98003447-2020	EPI-ISL-539256
hCoV-19-Spain-VC-IBV-98003489-2020	EPI-ISL-539281
hCoV-19-Spain-VC-IBV-98003490-2020	EPI-ISL-539282
hCoV-19-Spain-VC-IBV-98003492-2020	EPI-ISL-539283
hCoV-19-Spain-VC-IBV-98003494-2020	EPI-ISL-539284
hCoV-19-Spain-NC-CHN-01000315-2020	EPI-ISL-1383000
hCoV-19-Spain-NC-CHN-01000320-2020	EPI-ISL-1383005
hCoV-19-Spain-NC-CHN-01000321-2020	EPI-ISL-1383006
hCoV-19-Spain-NC-CHN-01000322-2020	EPI-ISL-1383007
hCoV-19-Spain-NC-CHN-01000325-2020	EPI-ISL-1383010
hCoV-19-Spain-NC-CHN-01000326-2020	EPI-ISL-1383011
hCoV-19-Spain-NC-CHN-01000327-2020	EPI-ISL-1383012
hCoV-19-Spain-NC-CHN-01000328-2020	EPI-ISL-1383013
hCoV-19-Spain-CT-IBV-003163-2020	EPI-ISL-510259
hCoV-19-Spain-GA-IBV-004041-2020	EPI-ISL-510280
hCoV-19-Spain-GA-IBV-004042-2020	EPI-ISL-510281
hCoV-19-Spain-GA-IBV-004046-2020	EPI-ISL-510285
hCoV-19-Spain-GA-IBV-004052-2020	EPI-ISL-510291
hCoV-19-Spain-GA-IBV-004053-2020	EPI-ISL-510292
hCoV-19-Spain-GA-IBV-004054-2020	EPI-ISL-510293
hCoV-19-Spain-GA-IBV-004055-2020	EPI-ISL-510294
hCoV-19-Spain-GA-IBV-004058-2020	EPI-ISL-510296
hCoV-19-Spain-GA-IBV-004060-2020	EPI-ISL-510298
hCoV-19-Spain-VC-FISABIO-77-2020	EPI-ISL-425201
hCoV-19-Spain-VC-FISABIO-79-2020	EPI-ISL-425203
hCoV-19-Spain-VC-FISABIO-86-2020	EPI-ISL-425210
hCoV-19-Spain-VC-FISABIO-96-2020	EPI-ISL-425220
hCoV-19-Spain-VC-FISABIO-97-2020	EPI-ISL-425221
hCoV-19-Spain-AN-IBV-002910-2020	EPI-ISL-467062
hCoV-19-Spain-CN-IBV-002605-2020	EPI-ISL-467089
hCoV-19-Spain-CT-IBV-003151-2020	EPI-ISL-510248
hCoV-19-Spain-CT-IBV-003154-2020	EPI-ISL-510251
hCoV-19-Spain-CT-IBV-003155-2020	EPI-ISL-510252
hCoV-19-Spain-CT-IBV-003164-2020	EPI-ISL-510260
hCoV-19-Spain-CT-IBV-003167-2020	EPI-ISL-510263
hCoV-19-Spain-VC-FISABIO-49-2020	EPI-ISL-420130
hCoV-19-Spain-VC-FISABIO-51-2020	EPI-ISL-420132
hCoV-19-Spain-AN-IBV-001089-2020	EPI-ISL-452597
hCoV-19-Spain-AN-IBV-001088-2020	EPI-ISL-452598
hCoV-19-Spain-GA-USC-009-2020	EPI-ISL-468339
hCoV-19-Spain-GA-USC-010-2020	EPI-ISL-468340
hCoV-19-Spain-CT-HUVH-GEST1-4-2020	EPI-ISL-728361
hCoV-19-Spain-CT-HUVH-GEST1-3-2020	EPI-ISL-728362
hCoV-19-Spain-CT-HUVH-GEST1-2-2020	EPI-ISL-728364
hCoV-19-Spain-CT-HUVH-GEST1-1-2020	EPI-ISL-728365
hCoV-19-Spain-NC-IBV-001398-2020	EPI-ISL-452503
hCoV-19-Spain-AN-IBV-001217-2020	EPI-ISL-452556

hCoV-19-Spain-AN-IBV-001196-2020	EPI-ISL-452562
hCoV-19-Spain-AN-IBV-001176-2020	EPI-ISL-452567
hCoV-19-Spain-AN-IBV-001104-2020	EPI-ISL-452586
hCoV-19-Spain-AN-IBV-001093-2020	EPI-ISL-452593
hCoV-19-Spain-PV-IBV-003260-2020	EPI-ISL-481004
hCoV-19-Spain-PV-IBV-003258-2020	EPI-ISL-481023
hCoV-19-Spain-MD-IBV-004940-2020	EPI-ISL-481074
hCoV-19-Spain-MD-IBV-004943-2020	EPI-ISL-481092
hCoV-19-Spain-MD-HGUGM-549606-2020	EPI-ISL-778645
hCoV-19-Spain-VC-FISABIO-52-2020	EPI-ISL-421519
hCoV-19-Spain-PV-IBV-000918-2020	EPI-ISL-452651
hCoV-19-Spain-AN-IBV-001080-2020	EPI-ISL-452605
hCoV-19-Spain-AN-IBV-001078-2020	EPI-ISL-452606
hCoV-19-Spain-PV-IBV-000953-2020	EPI-ISL-452622
hCoV-19-Spain-PV-IBV-000879-2020	EPI-ISL-452680
hCoV-19-Spain-PV-IBV-000874-2020	EPI-ISL-452683
hCoV-19-Spain-PV-IBV-000873-2020	EPI-ISL-452684
hCoV-19-Spain-CL-ISCIII-206192-2020	EPI-ISL-862567
hCoV-19-Spain-CT-ISCIII-209819-2020	EPI-ISL-862627
hCoV-19-Spain-CT-ISCIII-202034658-2020	EPI-ISL-862628
hCoV-19-Spain-CT-ISCIII-202034692-2020	EPI-ISL-862629
hCoV-19-Spain-CT-ISCIII-202034693-2020	EPI-ISL-862630
hCoV-19-Spain-CT-ISCIII-202034697-2020	EPI-ISL-862631
hCoV-19-Spain-CT-ISCIII-202034702-2020	EPI-ISL-862632
hCoV-19-Spain-CT-ISCIII-2017689-2020	EPI_ISL_8725398
hCoV-19-Spain-CT-ISCIII-2017690-2020	EPI_ISL_8725399
hCoV-19-Spain-CT-ISCIII-2017691-2020	EPI_ISL_8725400
hCoV-19-Spain-CT-ISCIII-2017692-2020	EPI_ISL_8725401
hCoV-19-Spain-CT-ISCIII-2017693-2020	EPI_ISL_8725402
hCoV-19-Spain-CT-ISCIII-2017694-2020	EPI_ISL_8725403
hCoV-19-Spain-CT-ISCIII-2017695-2020	EPI_ISL_8725404
hCoV-19-Spain-CT-ISCIII-2017696-2020	EPI_ISL_8725405
hCoV-19-Spain-CT-ISCIII-2017697-2020	EPI_ISL_8725406
hCoV-19-Spain-CT-ISCIII-2017698-2020	EPI_ISL_8725407
hCoV-19-Spain-CT-ISCIII-2017699-2020	EPI_ISL_8725408
hCoV-19-Spain-CT-ISCIII-2017700-2020	EPI_ISL_8725409
NC-045512.2 Severe acute respiratory syndrome coronavirus 2-isolate-Wuhan-Hu-1-*complete*-genome: GenBank accession number: NC_045512	

Supplementary Table S2. Summary of 12 samples of SARS-Cov-2 genetic study.

Sample number	Date of collection	Days since first sampling	Nucleotide mutations*	Amino acid mutations*	Completed by Sanger	Lineage
1	2020-03-24	0	7	2		B.1
2	2020-03-30	6	8	3		B.1
3	2020-04-28	35	8	3		B.1
4	2020-05-18	55	11	5	Yes	B.1
5	2020-06-02	70	12	6		B.1
6	2020-06-22	90	14	8		B.1
7	2020-07-30	128	20	15		B.1
8	2020-08-03	132	18	12	Yes	B.1
9	2020-08-07	136	16	10		B.1
10	2020-11-06	227	17	10		B.1
11	2020-11-12	233	21	13	Yes	B.1
12	2020-11-16	237	29	22		B.1

* Compared with the reference SARS-CoV-2 Wuhan-1 (GenBank accession number: NC 045512).

Supplementary Figure S3. Description of the different mutations in nsp3 dominions over time.

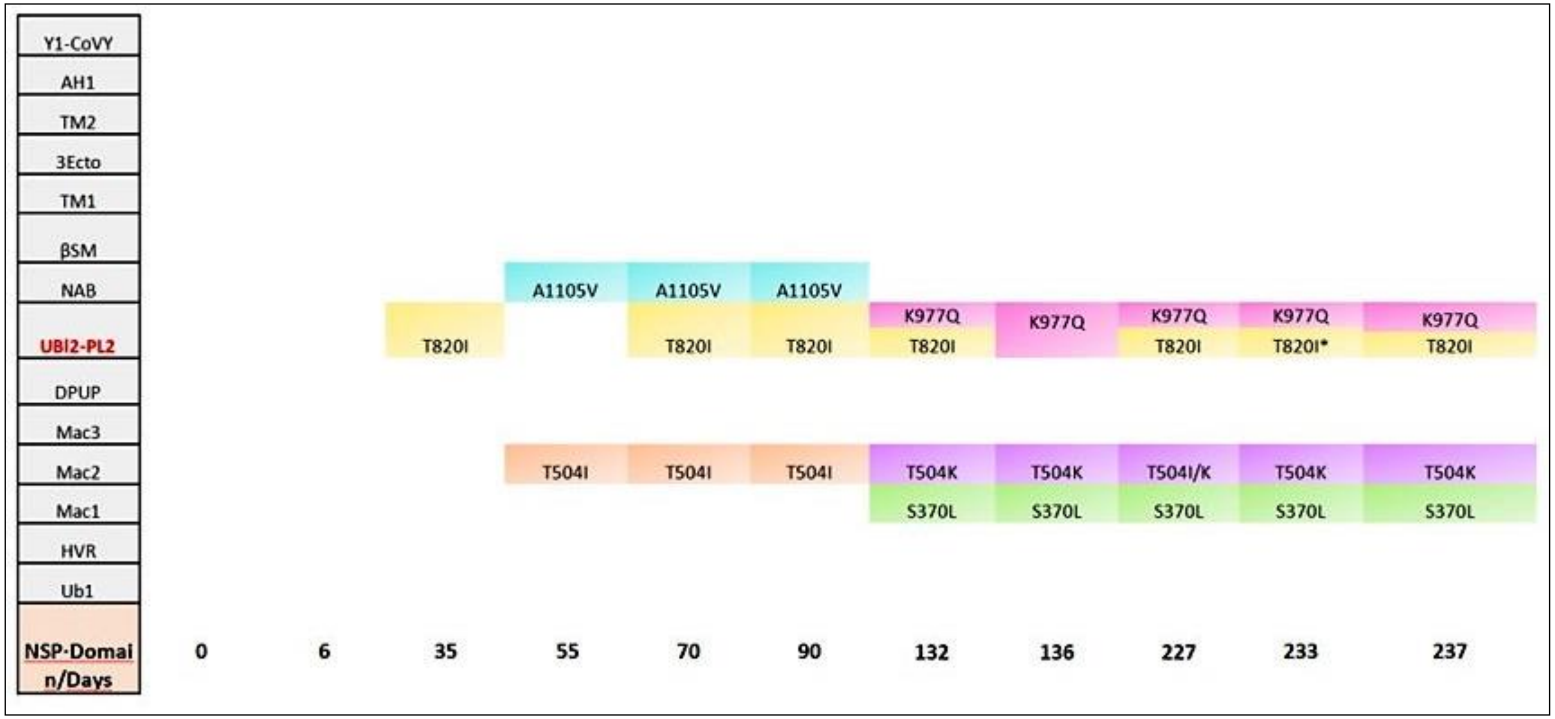

Legend. The X axis represents the days since the first positive sample. Minor variants and amino acid changes in nsp3 appeared after day 35. According to the consensus sequences, 6 amino acids changes were described over time in nsp3, mainly in Mac1, Mac 2 and UBl2-Pl2pro domains. The amino acid change S370L in Mac1 was detected in samples from day 132 to day 237. Mac2 presented two amino acid non-conservative changes in the position 504 of the protein. From day 55 to day 90 a change from tryptophan to interleucine was evidenced, and this interleucine that turned into a lysine from day 132 to 237. Three changes in the UBl2-Pl2pro domain were evidenced: T820I, K977Q and E746A.

Supplementary Figure S4. The most important mutations documented in the consensus sequences of the Spike protein over time.

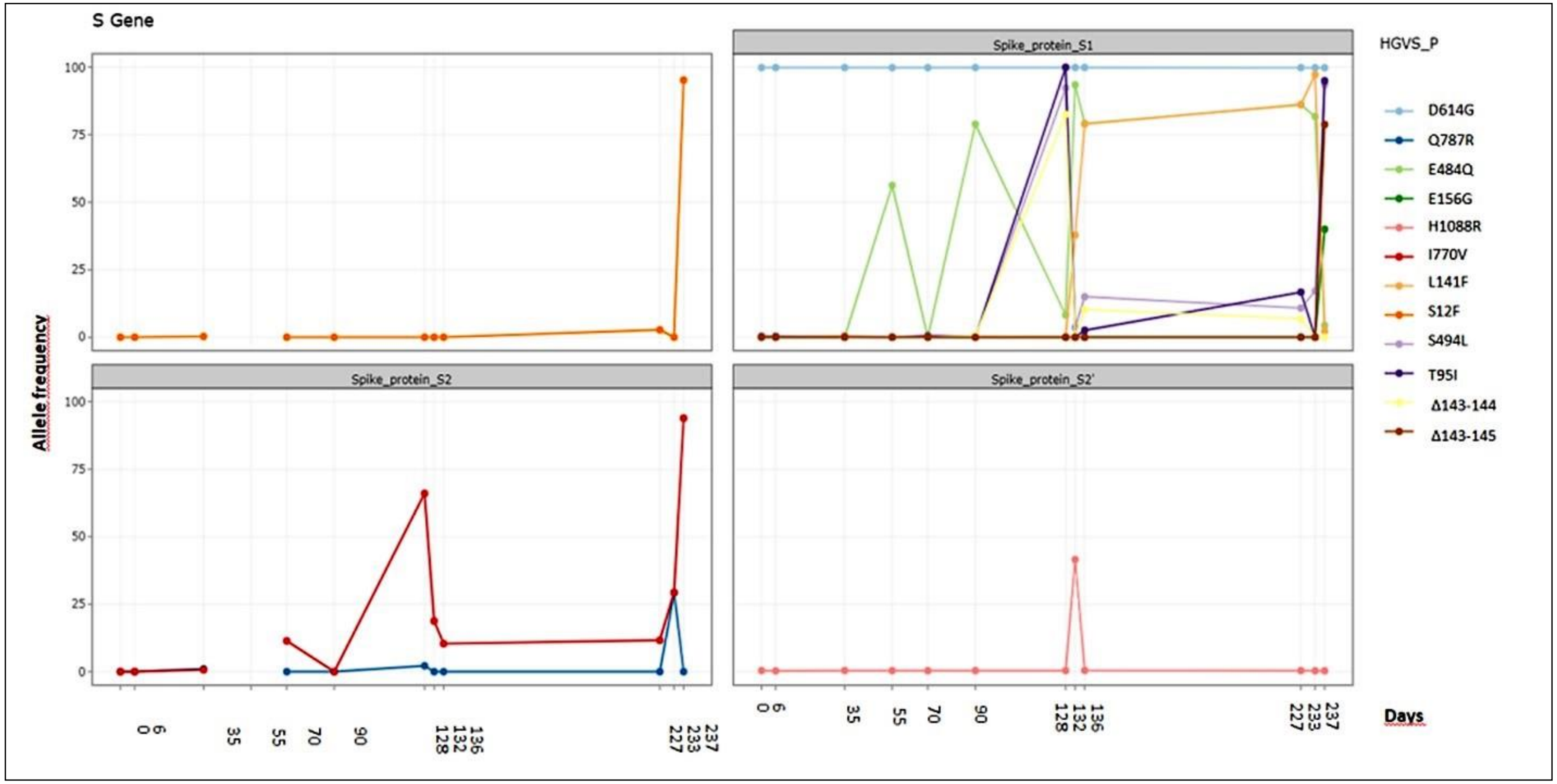

Legend. Time evolution of the most relevant mutations in the Spike protein. Variants showed a frequency from 0% to 100% (Y axis) over the different sampling dates (X axis). Amino acid substitutions are indicated by points using the same coloring associated to each name in the legend.

Supplementary Figure S5. Amino acid changes in the Spike protein observed in the last sample.

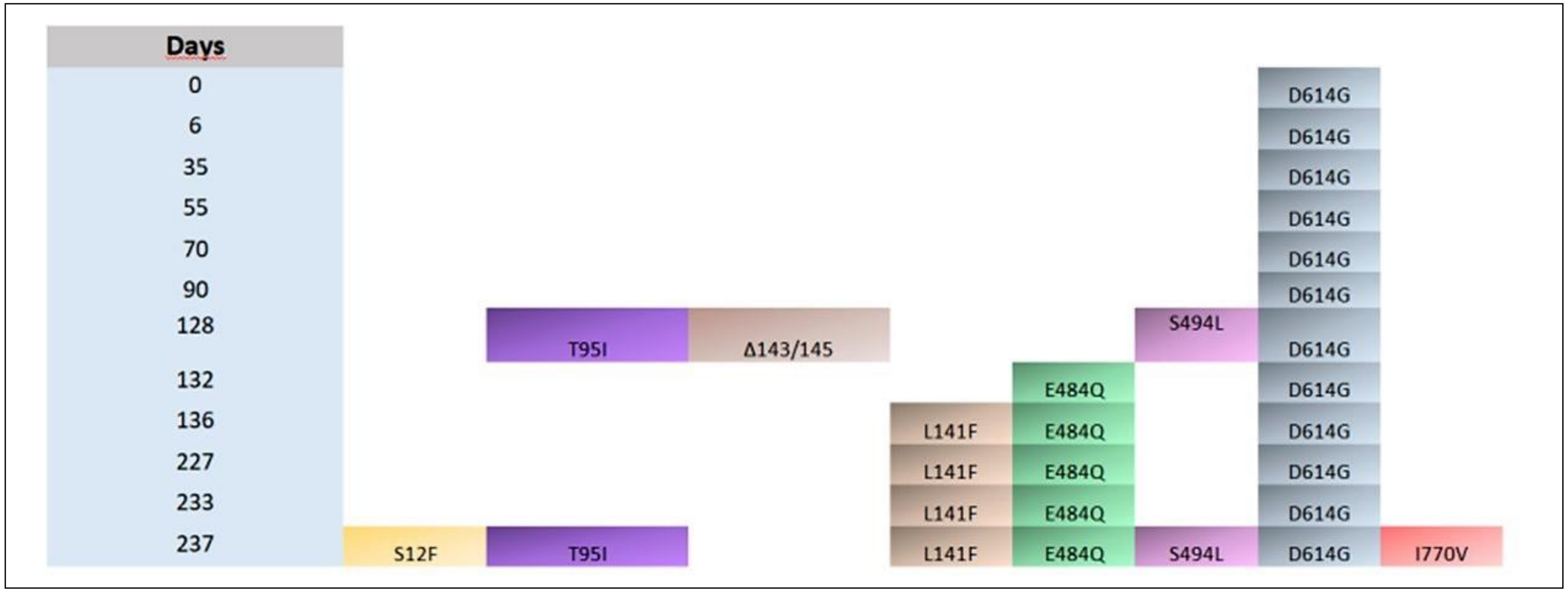

Legend. Description of the different mutations characterized in the Spike's consensus sequences over time. The X axis represents the days since the first positive sample. According to the consensus sequences, 8 amino acids changes were described over time in the Spike protein.
